# Exit-site infection and subsequent incident risk of peritoneal dialysis-associated peritonitis: a retrospective cohort study with meta-analysis

**DOI:** 10.3389/fpubh.2026.1893696

**Published:** 2026-08-12

**Authors:** Surapon Nochaiwong, Chidchanok Ruengorn, Kajohnsak Noppakun, Nuttaya Wachiraphansakul, Thanawat Vongchaiudomchoke, Tanun Ngamvichchukorn, Kednapa Thavorn, Manish M. Sood, Greg A. Knoll, Apichat Tantraworasin

**Affiliations:** 1 Department of Bioinformatic and Clinical Epidemiology, Faculty of Medicine, Chiang Mai University, Chiang Mai, Thailand; 2 Department of Pharmaceutical Care, Faculty of Pharmacy, Chiang Mai University, Chiang Mai, Thailand; 3 Pharmacoepidemiology and Statistics Research Center (PESRC), Faculty of Pharmacy, Chiang Mai University, Chiang Mai, Thailand; 4 Division of Nephrology, Department of Internal Medicine, Faculty of Medicine, Chiang Mai University, Chiang Mai, Thailand; 5 Department of Internal Medicine, Lampang Hospital, Lampang, Thailand; 6 Division of Nephrology, Department of Medicine, Faculty of Medicine Vajira Hospital, Navamindradhiraj University, Bangkok, Thailand; 7 Ottawa Hospital Research Institute, Ottawa Hospital, Ottawa, ON, Canada; 8 School of Epidemiology and Public Health, Faculty of Medicine, University of Ottawa, Ottawa, ON, Canada; 9 Institute of Clinical and Evaluative Sciences, ICES uOttawa, Ottawa, ON, Canada; 10 Division of Nephrology, Department of Medicine, University of Ottawa, Ottawa, ON, Canada; 11 Department of Surgery, Faculty of Medicine, Chiang Mai University, Chiang Mai, Thailand; 12 Clinical Surgical Research Center, Chiang Mai University, Chiang Mai, Thailand

**Keywords:** catheter-related infection, exit-site infection, peritoneal dialysis, peritonitis, retrospective cohort, systematic review and meta-analysis

## Abstract

**Background:**

To date, evidence to support the risk estimates of peritoneal dialysis (PD)-associated peritonitis after the occurrence of exit-site infection (ESI) is limited. We aimed to investigate the association between ESI and the subsequent risk of PD-associated peritonitis.

**Methods:**

We conducted a multicenter, retrospective cohort study of adult incident PD patients in Thailand between January 2006 and September 2022. We compared matched pairs (1:1) of individuals who experienced the first episode of ESI with their counterparts who did not experience any ESIs during follow-up. The subsequent risk of PD-associated peritonitis was assessed using multivariable Cox proportional hazards models with shared frailty correction to estimate hazard ratios (HRs) and 95% confidence intervals (CIs). Landmark analysis corresponding to the designed landmark time after ESI was conducted (within 1 month, 1–3 months, 3–6 months, 6–9 months, and 9–12 months). Secondary analyses explored causative organisms and ESI severity. An ancillary meta-analysis of all available evidence was summarized using a random-effect model.

**Results:**

Among 5,368 incident PD cases in the cohort, a total of 610 patients were eligible, including 305 incident cases of the first episode of ESI and 305 corresponding matched index cases. The study cohort had a mean age of 59.3 years; most patients were male (56.7%), and most received continuous ambulatory PD (89.0%). Regarding adjustment for sociodemographic and clinical characteristics, PD practices, and laboratory profiles, the risk of subsequent PD-associated peritonitis was significantly increased among individuals who had experienced an ESI (HR, 5.30; 95% CI, 3.75–7.50). Following landmark analysis, the risk of PD-associated peritonitis increased at peak approximately 18-fold (within 1 month) and gradually decreased to 2-fold over the designed 9–12 months. Secondary analyses revealed consistent findings across the causative organism and severity strata of ESIs (HRs ranging from 5.17 to 5.98). An ancillary meta-analytic synthesis supported the main findings [3 included cohort studies; *n* = 1775; HR, 3.37 (95% CI, 1.35–8.41)].

**Conclusion:**

Regarding retrospective cohort analysis with ancillary meta-analytical synthesis, individuals experienced with ESI are at risk of developing PD-associated peritonitis. Effective strategies to prevent PD-associated peritonitis following an ESI episode are warranted to promote patient health and sustainable PD programs.

## Introduction

Peritoneal dialysis (PD) is recognized as a home dialysis option for patients with end-stage kidney disease (ESKD) (1). As of today, PD programs have been adopted and are available in healthcare services in over 79% of countries worldwide, particularly in resource-limited healthcare settings (2). Regarding heterogeneity in healthcare services and policy allocation, approximately 21.0 per million population have been treated with PD as a long-term kidney replacement therapy (2). To date, evidence suggests that PD modalities offer potential advantages, for instance, better physical function outcomes and health-related quality of life, good monetary value due to lower cost of healthcare utilization, and better outcomes during the transition or waiting list and after kidney transplantation, compared with chronic hemodialysis (1, 3, 4).

Despite improvements in PD practice and treatment, and innovations in PD modalities over the past several decades, infectious complications (i.e., catheter-related infections and PD-associated peritonitis) remain a major concern for PD treatment in contemporary nephrology practice (1, 5, 6). A pathological concept has been hypothesized that exit-site infection (ESI) may be associated with subsequent PD-associated peritonitis via translocation of microorganisms from the exit site into the peritoneal cavity through periluminal movement along the PD catheter path (3, 7–11). The increased risk is especially noticeable with Gram-positive causative microorganisms, particularly *Staphylococcus aureus* and Coagulase-negative *Staphylococcus* (CoNS) (12).

With respect to limited clinical epidemiology studies available (12–14), there are several compelling reasons to reevaluate and expand analysis to the previous evidence: (i) existing studies being limited to residual confounders in relation to PD-associated peritonitis (e.g., key traditional well recognized risks such as serum albumin), (ii) studies being restricted to nondiverse PD populations; (iii) the analyses included prevalent or mixed cases PD resulting in some studies may include individuals who had a history of PD-associated peritonitis before the first episode of ESI, which may profound and not accurately reflect the likelihood of the findings, and (iv) the evidence being based on studies published before 2015, which may not reflect update international guideline recommendations for prevention and treatment of catheter-related infections in current practice.

Given this knowledge gap, we conducted a multicenter, retrospective cohort study of the adult Thai PD population, comparing matched pairs of individuals who experienced the first episode of ESI with corresponding individuals without any episode of ESI, and the subsequent risk of PD-associated peritonitis. Given the limited available evidence, we then combined these findings from the cohort analysis with existing studies as part of a meta-analytic synthesis to contextualize and yield evidence-based conclusions.

## Methods

### Study design, data sources, and patient population

In this study, we conducted a multi-PD center, retrospective cohort study of newly adult incident PD populations in Chiang Mai, Thailand. We assembled data sources based on the Thai Renal Outcomes Research-Peritoneal Dialysis (THOR-PD) database (details are described elsewhere) (15–18). In brief, this database collected consecutively ESKD patients who newly initiated PD in 3 centers in Chiang Mai, Thailand. In this analysis, we merged the information, including (i) the routine collected data based on electronic health records, healthcare claims database that consists of outpatient and inpatient treatment information; (ii) the local laboratory support system extract that consists of claims laboratory information, including details of microbiology information for ESI and PD-associated peritonitis; and (iii) routine collected data of local PD patient care database that consists of individual patient-level sociodemographic and PD treatment care.

In this analysis, we included adult ESKD patients aged ≥18 years who commenced newly PD treatment, either continuous ambulatory PD (CAPD) or automated PD (APD), between January 1, 2006, and December 31, 2020, and were followed until September 30, 2022. We excluded individuals who had: (i) received PD treatment for acute kidney injury; (ii) experienced ESI or pre-PD peritonitis within 30 days following PD catheter insertion due to infections in this timeframe are likely associated with surgical procedure (known as PD catheter insertion-related exit-site and/or tunnel infection) (9, 11, 19); (iii) experienced with PD-associated peritonitis or tunnel infection before or on the same day of ESI to minimized reverse causal relationship and to establish a more homogeneous study population at risk of PD-associated peritonitis following the first episode of ESI; and (iv) incomplete follow-up information, and relevant data on ESI and PD-associated peritonitis.

All PD patients in this cohort received antibiotics at the time of PD catheter insertion to minimize the risk of early PD-associated peritonitis after PD program initiation (15, 16). Concerning long-term antibiotic resistance, all PD patients received a standard protocol of daily catheter exit-site care with a sterile normal saline dressing. Decolonization with 2% mupirocin ointment was applied to only patients identified as *S. aureus* nasal carriers before PD catheter insertion. Standard antibiotic regimens for the treatment of ESI and PD-associated peritonitis were based on the local PD center protocol, which was in line with the International Society for Peritoneal Dialysis (ISPD) recommendations (9, 19). Moreover, all PD patients and their primary caregivers received a standardized PD training program before PD initiation, including sterile techniques for PD bag exchange.

This study obtained ethical approval from the institutional review boards of the Faculty of Medicine, Chiang Mai University (No. 428/2021), and Nakornping Hospital (No. 076/64), Chiang Mai, Thailand. Given the nature of the retrospective cohort study design, informed consent was not required, as the data were anonymized and de-identified. We reported the study according to the Strengthening the Reporting of Observational Studies in Epidemiology statements (20).

### Exposure

The exposure group for this study comprised individuals diagnosed with ESI according to the 2023 ISPD recommendations for catheter-related infection (19), as verified by two independent investigators (SN and KN). Definitive ESI was defined as the presence of purulent discharge at the catheter site, with or without erythema (19). Individuals who experienced only signs of inflammation at the exit-site or positive culture in equivocal cases without purulent discharge were excluded from this analysis. Individuals without any ESI episodes were classified as the non-exposure group.

### Covariates

We derived covariate information during the prelude period, defined as the period between the PD initiation (post-break-in period) and the date of diagnosis with the first episode of ESI for the exposure case, and the time-matched index date for the corresponding non-exposure case. The relevant variable included (i) patient sociodemographic [i.e., age, sex, body mass index (BMI), marital status, working status, educational level, smoking status, alcohol drinking status, reimbursement scheme, and living distance from PD center], (ii) clinical characteristics and PD practices [i.e., year of PD initiation, PD centers, estimated glomerular filtration rate (eGFR) at PD initiation, urgent-start PD (based on catheter break-in period ≤14 days), PD modality (CAPD and APD), residual kidney function at PD initiation (defined as urine output ≥200 mL/day), etiology of ESKD, Charlson comorbidity index (non-age adjusted), and comorbidities (diabetes mellitus, coronary artery disease, chronic heart failure, cerebrovascular disease)], and (iii) laboratory profiles (i.e., serum creatinine, serum urea nitrogen, serum albumin, hemoglobin, ferritin, transferrin saturation, sodium, potassium, bicarbonate, calcium, phosphorus, intact parathyroid hormone, neutrophil-to-lymphocyte ratio, platelet-to-lymphocyte ratio, and alkaline phosphatase).

### Outcome: PD-associated peritonitis

The outcome of interest was the episode of PD-associated peritonitis, which was defined according to the 2022 ISPD criteria (9) and verified by two independent investigators (SN and KN). The occurrence of PD-associated peritonitis was identified based on at least 2 criteria of the following: (i) clinical features consistent with peritonitis (i.e., abdominal pain and/or cloudy dialysis effluent); (ii) dialysis effluent white blood cell count >100 cells/μL, in which >50% are polymorphonuclear leukocytes; and (iii) positive dialysate effluent culture (9).

### Statistical analysis

Regarding previous studies (12, 14), the adjusted hazard ratios (HRs) for PD-associated peritonitis following ESI ranged from 1.59 to 4.68, with median follow-up of 18 to 19 months and peritonitis-free rates of 61.9 to 83.2%. Considering a two-tailed level of 0.05 and 90% power, a minimum sample size of 444 eligible PD patients (222 per exposure and non-exposure groups) is needed for this study. Of these, 204 expected cases of PD-associated peritonitis were needed to detect an increased risk of PD-associated peritonitis following the first episode of ESI compared to individuals without any episode of ESI.

To investigate the risk of PD-associated peritonitis following the first episode of ESI, individuals who experienced ESI (exposure group) were matched 1:1 to individuals without any episode of ESI based on age (within 1 year), sex, and prelude period (within 1 month), defined as the period between PD initiation and the date of the first episode of ESI (index date or inception time of cohort) for the exposure group or time-matched prelude period for the non-exposure group. Based on time-to-event analysis, the outcomes of interest were identified after the index date (date of diagnosis with ESI for exposure cases or time-matched prelude period for non-exposure cases), and follow-up ended on September 30, 2022 (study end date). Censored data were employed in case of transfer to other dialysis centers, PD discontinuation (either kidney transplantation or switched to hemodialysis due to PD technical failure), loss to follow-up, or death, whichever comes first.

All analyses and graphical visualization were performed using Stata version 18.5 (College Station, Texas, United States). Two-tailed tests with a *p*-value <0.05 were considered statistically significant. We considered a complete case analysis given the low rate of missing data (0.7–2.1%; see details in Supplementary Table S1). Descriptive analysis was employed, reported as numbers (percentages) and mean (standard deviation [SD]) or median (min-max), as appropriate. For the main analysis, we employed shared frailty correction in Cox proportional hazards models to investigate the subsequent risk of PD-associated peritonitis after the first ESI episode (18, 21, 22). The assumption of proportional hazards was appropriately explored and tested using Schoenfeld residuals plots. Multivariable hierarchical Cox regression analysis with a series of consecutive 3 model degree adjustments of covariates as described was employed as follows: (i) model 1, which consists of patients’ sociodemographic characteristics, (ii) model 2, which consists of model 1 plus clinical characteristics and PD practices, and (iii) model 3 (full model), which consists of model 2 plus laboratory profiles. We explored the multicollinearity assumption for *a priori* covariates included in the model adjustment before formal analysis.

To account for immortal time bias and address clinical interpretation, we employed a set of landmark analyses (the landmark time) based on the literature as follows: within 1 month, 1–3 months, 3–6 months, 6–9 months, and 9–12 months after the first episode of ESI (12, 14). Only individuals who survived or remained receiving PD treatment until the designated landmark time were considered in the analysis. For secondary analysis, different approaches to categorize the exposure were selected a priori according to clinical relevance: (i) causative organism of ESI (i.e., gram-positive or gram-negative), specific common pathologic organism of ESI [*S. aureus* or CoNS], (ii) severity strata of causative organism of ESI (low, moderate, and high risk of mortality or catheter removal rate (16), see details in Supplementary Table S2). Sensitivity analyses were performed to explore the robustness of the findings: (i) *post hoc* analysis based on Cox proportional hazards models without shared frailty correction, (ii) reanalysis by excluding individuals who experienced ESI with culture-negative results, and (iii) estimating the expected (E)-value to explore the impact of residual confounders of the full model adjustments in observational studies (23). The effect estimates were reported as HRs with 95% confidence intervals (CIs).

To support the main analysis and yield more evidence-based conclusions, we also employed an ancillary analysis using a standard pairwise systematic review and meta-analysis that incorporates existing literature evidence alongside the main findings from this cohort analysis. The predefined protocol was prospectively registered in the International Prospective Register of Systematic Reviews (CRD42018105483). As part of the supplemented analysis, we followed the Preferred Reporting Items for Systematic Reviews and Meta-Analyses reporting statement (24). Details of methods and protocol amendment for this additional systematic review are described in Supplementary Appendix S1. In brief, we systematically searched 6 electronic databases, including grey literature, to identify all published articles that investigated the risk of subsequent PD-associated peritonitis following ESI, from inception to May 25, 2026, without language restrictions. To minimize methodological heterogeneity, only cohort studies that reported adjusted HRs were eligible for this meta-analysis. The quality of each included cohort study was assessed using the Newcastle-Ottawa Scale (NOS) (25) by two reviewers (SN and CR) independently.

Regarding the meta-analytic approach, we pooled the adjusted HRs with 95% CI using random-effects models to account for within and between-study variance across the included studies (26). We also estimated 95% prediction intervals to account for the predicted range, which addresses the expected uncertainty of the summary pooled effect estimates (27). The certainty of the evidence findings was appraised and classified as insufficient, very low, low, moderate, or high quality (4, 28, 29). With respect to contextualized approaches of evidence-based conclusions, the pooled association was then judged as trivial/neutral (not substantially different from that of the individuals without any episode of ESI), beneficial (protective effect or decreased risk of PD-associated peritonitis), or harmful (increased risk of PD-associated peritonitis) (4, 30).

## Results

### Description of patients included in the cohort

**Figure 1:**
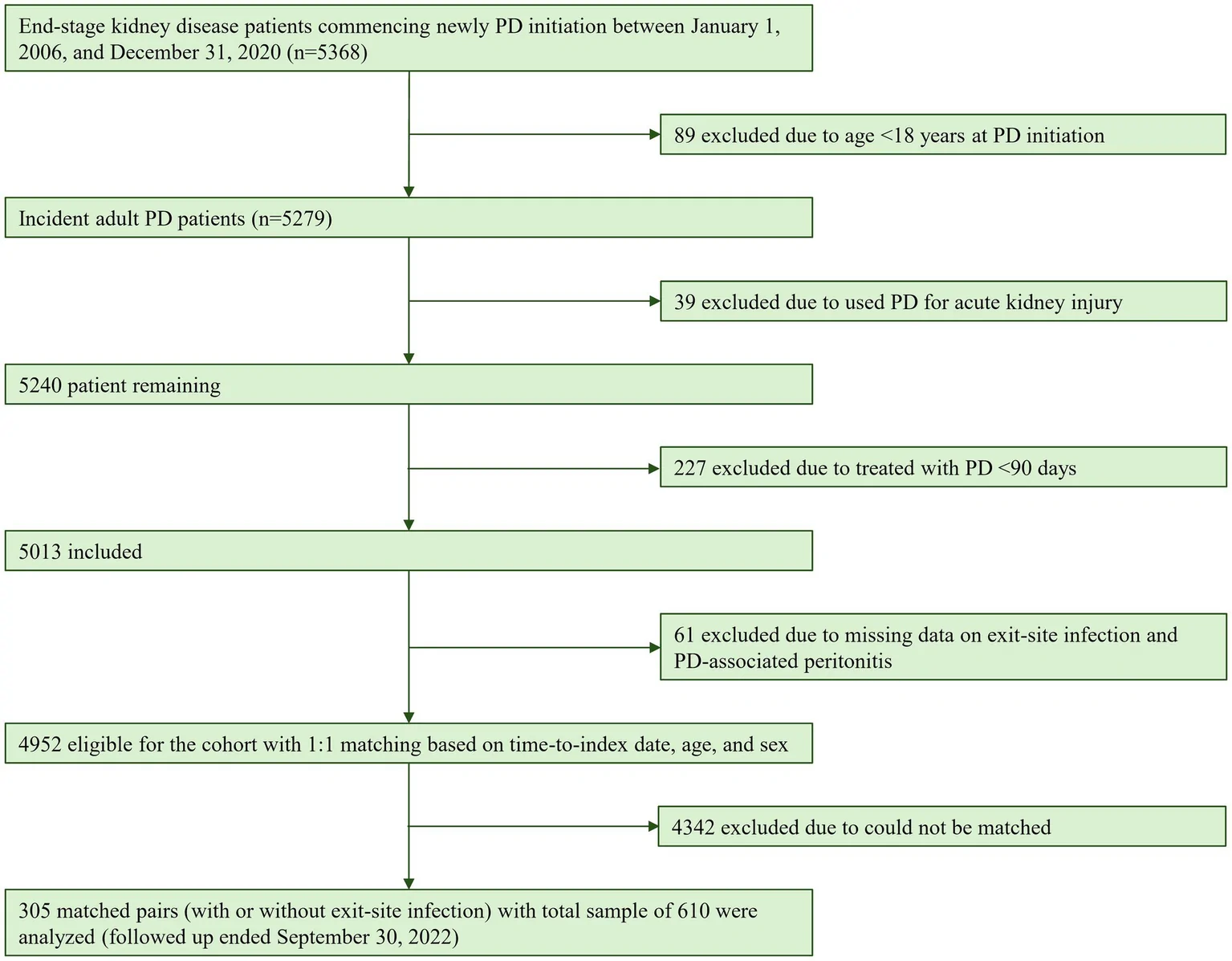
Study flow on the selection of eligible patients for the THOR-PD cohort analysis. PD, peritoneal dialysis; THOR-PD, Thai Renal Outcomes Research-Peritoneal Dialysis.

Among the 5,368 incident PD cases screened, 4,951 were eligible for the cohort, with 1:1 matching based on age, sex, and time-to-index date (Figure 1). Of these, 305 matched pairs (305 for each group of individuals with and without any episode of ESI) were included in this analysis. During the PD treatment, the study sample had a median survival of 8.0 years, with a total of 2540.9 patient-years of follow-up (Supplementary Table S3).

Overall, the cohort samples were comparable based on the matching variables. The cohort sample had a mean age (SD) of 59.3 (10.7), 346 were male (56.7%), and had a median time-to-index date of 8.5 months after PD initiation (Table 1). Moreover, most PD patients were treated with CAPD, with a difference between individuals with and without ESI (92.5% vs. 85.6%, *p* = 0.009; Table 1). Several covariates, including patient sociodemographic and clinical characteristics, PD practices, and laboratory profiles, were generally different between individuals who experienced the first episode of ESI and their matched counterparts without any ESI episode. For example, individuals who experienced ESI had a lower educational level, more comorbid conditions, and poorer proxies of nutritional status [e.g., lower BMI and serum albumin, and higher biomarkers of systemic immune-inflammation index (e.g., neutrophil-to-lymphocyte ratio)]. Table 1 describes patient characteristics of 305 matched pairs included in this analysis.

**Table 1:** Patient characteristics of 305 matched pairs in the THOR-PD cohort^a^.

Patient characteristics	Total eligible sample (*n* = 610)	Matched Individuals		*p* value
		Without ESI group (*n* = 305)	With ESI group (*n* = 305)	
Matching variables				
Age (years)	59.3 (10.7)	59.3 (10.7)	59.2 (10.7)	0.947
<65	439 (72.0)	219 (71.8)	220 (71.1)	1.000
65–75	124 (20.3)	62 (20.3)	62 (20.3)	
>75	47 (7.7)	24 (7.9)	23 (7.5)	
Male sex	346 (56.7)	173 (56.7)	173 (56.7)	1.000
Time-to-index date (month), median (min—max) (time-matched 1:1)^b^	8.5 (1.2–21.8)	8.6 (1.2–21.8)	8.5 (1.3–21.8)	0.977
Sociodemographic				
BMI (kg/m^2^)	22. 0 (3.4)	22.3 (3.4)	21.7 (3.4)	0.038
<18.5	90 (14.8)	37 (12.1)	53 (17.4)	0.135
18.5–22.9	310 (50.8)	155 (50.8)	155 (50.8)	
≥23.0	210 (34.4)	113 (37.1)	97 (31.8)	
Marital status				
Single	64 (10.5)	29 (9.5)	35 (11.5)	0.532
Married	464 (76.1)	238 (78.0)	226 (74.1)	
Widow/divorced/other	82 (13.4)	38 (12.5)	44 (14.4)	
Unemployed/retried	166 (27.2)	83 (27.2)	83 (27.2)	1.000
Educational level				
Illiterate/primary school	468 (76.7)	218 (71.5)	250 (82.0)	0.009
Junior to high school	66 (10.8)	40 (13.1)	26 (8.5)	
Diploma/bachelor/higher	76 (12.5)	47 (15.4)	29 (9.5)	
Current smoker^c^	27 (4.4)	16 (5.2)	11 (3.6)	0.432
Current alcohol drinking^c^	15 (2.5)	8 (2.6)	7 (2.3)	1.000
Reimbursement scheme				
National health security office	471 (77.2)	224 (73.4)	247 (81.0)	0.072
Civil servant medical benefit	115 (18.9)	68 (22.3)	47 (15.4)	
Others	24 (3.9)	13 (4.3)	11 (3.6)	
Living distance ≥100 km from PD center	147 (24.1)	74 (24.3)	73 (23.9)	1.000
Clinical characteristics and PD practices				
Year of PD initiation				
2006–2010	144 (23.6)	66 (21.6)	78 (25.6)	0.050
2011–2015	312 (51.2)	149 (48.9)	163 (53.4)	
2016–2020	154 (25.2)	90 (29.5)	64 (21.0)	
eGFR at PD initiation (mL/min/1.73 m^2^)	6.2 (3.1)	6.2 (3.0)	6.3 (3.1)	0.571
Early start (≥10)	81 (13.3)	35 (11.5)	46 (15.1)	0.387
Intermediate start (5–9.9)	274 (44.9)	142 (46.5)	132 (43.3)	
Late start (<5)	255 (41.8)	128 (42.0)	127 (41.6)	
Urgent-start PD (catheter break-in period ≤14 days)	74 (12.1)	18 (5.9)	56 (18.4)	<0.001
PD modality				
CAPD	543 (89.0)	261 (85.6)	282 (92.5)	0.009
APD	67 (11.0)	44 (14.4)	23 (7.5)	
Residual kidney function at PD initiation (urine output ≥200 mL/day)	476 (78.0)	244 (80.0)	232 (76.1)	0.282
Etiology of ESKD				
Hypertensive nephrosclerosis	245 (40.2)	113 (37.1)	132 (43.3)	0.186
Diabetic nephropathy	197 (32.3)	97 (31.8)	100 (32.8)	
Glomerulonephritis	29 (4.7)	18 (5.9)	11 (3.6)	
Unknown/others	139 (22.8)	77 (25.2)	62 (20.3)	
Charlson comorbidity index (non-age adjusted), median (min—max)	3 (2–12)	3 (2–10)	4 (2–12)	0.008
Low (<5)	314 (51.5)	171 (56.1)	143 (46.9)	0.010
Medium (4—6)	254 (41.6)	121 (39.7)	133 (43.6)	
High (>6)	42 (6.9)	13 (4.3)	29 (9.5)	
Comorbid conditions				
Diabetes mellitus	256 (42.0)	119 (39.0)	137 (44.9)	0.163
Coronary artery disease	60 (9.8)	33 (10.8)	27 (8.8)	0.497
Chronic heart failure	39 (6.4)	16 (5.2)	23 (7.5)	0.321
Cerebrovascular disease	25 (4.1)	12 (3.9)	13 (4.3)	1.000
Laboratory profiles				
Serum creatinine (mg/dL)	9.0 (3.7)	9.2 (3.6)	8.8 (3.8)	0.212
Serum urea nitrogen (mg/dL)	58.7 (24.4)	61.2 (24.8)	56.2 (23.8)	0.012
Serum albumin (g/dL)	3.4 (0.6)	3.9 (0.3)	3.0 (0.5)	<0.001
Hemoglobin (g/dL)	9.9 (1.5)	10.1 (1.4)	9.7 (1.6)	0.001
Ferritin (ng/mL), median (min—max)	559.0 (25.5–2210.4)	519.0 (25.5–2210.4)	587.0 (76.0–2120.0)	0.001
Transferrin saturation, %	26.6 (4.8)	26.2 (3.7)	27.0 (5.7)	0.060
Sodium (mEq/L)	138.2 (3.9)	138.3 (3.5)	138.1 (4.2)	0.501
Potassium (mEq/L)	3.7 (0.8)	3.9 (0.7)	3.5 (0.8)	<0.001
Bicarbonate (mEq/L)	27.2 (4.0)	27.9 (3.9)	26.4 (4.0)	<0.001
Calcium (mg/dL)	9.0 (1.1)	9.0 (1.0)	8.9 (1.1)	0.452
Phosphorus (mg/dL)	4.3 (1.5)	4.3 (1.4)	4.3 (1.5)	0.935
Intact parathyroid hormone (pg/mL), median (min—max)	356.7 (11.9–1468.0)	329.4 (36.2–1277.8)	370.1 (11.9–1468.0)	0.412
Neutrophil-to-lymphocyte ratio	2.8 (1.0)	2.6 (0.9)	3.0 (1.0)	<0.001
Platelet-to-lymphocyte ratio	152.7 (65.6)	148.3 (59.5)	157.2 (71.0)	0.094
Alkaline phosphatase (U/L), median (min—max)	95 (19–422)	83 (19–422)	105 (27–401)	<0.001

^a^Categorical variables are expressed as a number (percentage); continuous variables as mean (SD), unless otherwise indicated.

^b^The time from PD initiation to the first episode of exit-site infection was matched to that of individuals without any exit-site infection.

^c^Defined as those who were current smokers (drinkers) or smokers (drinkers) for at ≥6 months in the past and quit smoking (drinking) < 12 months ago.

APD, automated peritoneal dialysis; BMI, body mass index; CAPD, continuous ambulatory peritoneal dialysis; eGFR, estimated glomerular filtration rate; ESI, exit-site infection; ESKD, end-stage kidney disease; PD, peritoneal dialysis; SD, standard deviation; THOR-PD, Thai renal outcomes research-peritoneal dialysis.

### Risk of PD-associated peritonitis after ESI

**Figure 2:**
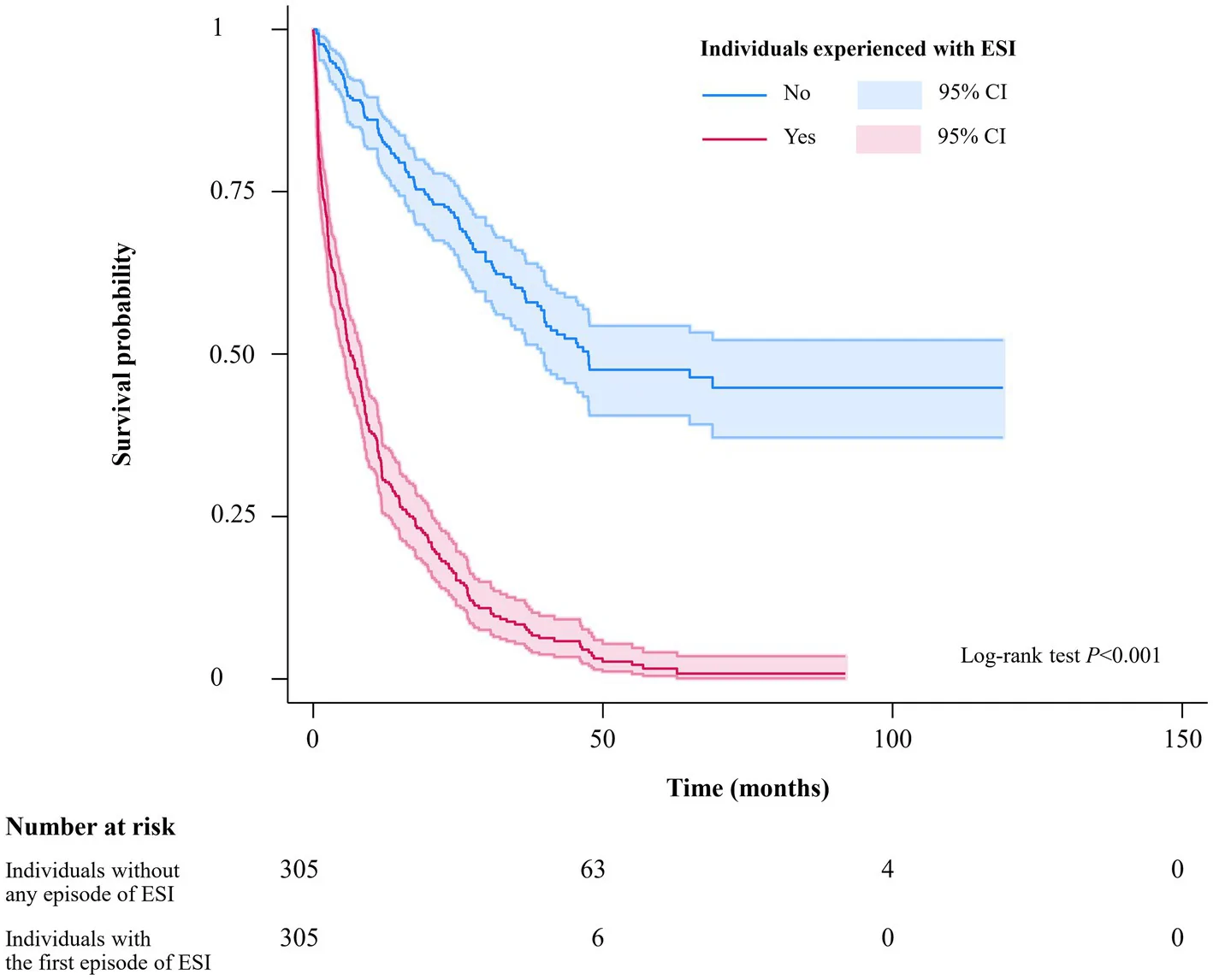
Kaplan–Meier plot for PD-associated peritonitis over time among individuals with and without ESI. Shaded areas represent 95% CI. CI, confidence interval; ESI, exit-site infection; PD, peritoneal dialysis.

In a time-matched analysis, 414 (67.9%) patients developed PD-associated peritonitis during a median follow-up of 1.5 years, for a total of 1107.7 patient-years at risk (Supplementary Table S3). Individuals experienced with the first episode of ESI had a higher likelihood of PD-associated peritonitis than individuals without any episode of ESI [crude absolute risk difference of 59.4% (95% CI, 53.3–65.5), Supplementary Table S3]. Figure 2 illustrates unadjusted Kaplan–Meier plots for PD-associated peritonitis in individuals with and without ESI (log-rank test, *p* < 0.001).

For multicollinearity testing, all covariates had variance inflation factor values <2.5 and tolerance values >0.40, indicating no collinearity concern. Moreover, Schoenfeld residual-based tests indicated that the proportional hazards assumption for the outcome of interest was not violated (*p* = 0.072). For the main analysis with shared frailty correction (Table 2), individuals experienced with the first episode of ESI had a higher subsequent risk of PD-associated peritonitis (full model adjusted HR, 5.30; 95% CI, 3.75–7.50); *p* < 0.001). Following the landmark analysis (Table 2), risk of PD-associated peritonitis after ESI were all statistically significant in all designed landmark time, which gradually decreased over time: full adjusted HR of 18.15 (95% CI, 6.76–40.27) for with 1-month, 10.44 (95% CI, 2.98–17.21) for 1–3 month, 6.79 (95% CI, 3.77–15.29) for 3–6 months, 4.19 (95% CI, 2.78–8.18) for 6–9 months, and 2.11 (95% CI, 1.62–7.89) for 9–12 months, with all *p* < 0.001. All effect estimates were consistent across multivariable hierarchical covariate adjustment and unadjusted analyses (Table 2 and Supplementary Table S5).

**Table 2:** Risk of PD-associated peritonitis among individuals with ESI in the 305 matched pairs THOR-PD cohort.

Outcome analyses (reference: individuals without exit-site infection)	PD-associated peritonitis		HR (95% CI): model 1^a^	*P* value	HR (95% CI): model 2^b^	*P* value	HR (95% CI): model 3 (full model)^c^	*P* value
	Events, No. (%)/total sample	Incidence rate per 100 patient-years (95% CI)						
Main analysis^d^								
Overall risk of PD-associated peritonitis after the first episode of ESI	414 (67.9)/610	3.1 (2.8–3.4)	7.80 (6.09–10.00)	<0.001	6.64 (5.17–8.54)	<0.001	5.30 (3.75–7.50)	<0.001
Landmark analysis^d^								
Within 1 month after ESI	65 (10.7)/610	11.1 (8.7–14.2)	48.21 (17.38–133.74)	<0.001	27.93 (9.12–85.48)	<0.001	18.15 (6.76–40.27)	<0.001
1–3 months after ESI	55 (9.0)/610	3.1 (2.4–4.0)	26.39 (10.40–66.98)	<0.001	15.14 (3.94–37.01)	<0.001	10.44 (2.98–17.21)	<0.001
3–6 months after ESI	60 (9.8)/610	1.7 (1.3–2.2)	4.53 (2.06–9.95)	<0.001	5.23 (2.25–12.15)	<0.001	6.79 (3.77–15.29)	<0.001
6–9 months after ESI	42 (6.9)/610	0.8 (0.6–1.1)	3.11 (1.72–5.60)	<0.001	3.46 (1.76–6.81)	<0.001	4.19 (2.78–8.18)	<0.001
9–12 months after ESI	40 (6.6)/610	0.6 (0.4–0.8)	1.76 (1.11–5.74)	<0.001	1.93 (1.45–6.74)	<0.001	2.11 (1.62–7.89)	<0.001
Secondary analysis^d^								
According to causative organism of ESI								
Gram-positive	185 (30.3)/610	7.7 (6.6–8.9)	7.55 (5.73–9.94)	<0.001	6.44 (4.90–8.47)	<0.001	5.24 (3.65–7.52)	<0.001
Gram-negative	80 (13.1)/610	10.4 (8.3–13.0)	7.89 (5.57–11.18)	<0.001	7.00 (4.96–9.86)	<0.001	5.35 (3.54–8.07)	<0.001
According to common pathologic organism of ESI								
*S. aureus*	101 (16.6)/610	7.5 (6.1–9.2)	7.74 (5.55–10.79)	<0.001	7.04 (5.07–9.76)	<0.001	5.65 (3.72–8.58)	<0.001
CoNS	56 (9.2)/610	9.5 (7.3–12.4)	8.27 (5.52–12.38)	<0.001	6.52 (4.44–9.57)	<0.001	5.45 (3.54–8.40)	<0.001
According to severity strata of causative organism of ESI								
Low	205 (33.6)/610	7.9 (6.8–9.1)	7.64 (5.84–9.99)	<0.001	6.50 (4.97–8.51)	<0.001	5.21 (3.64–7.46)	<0.001
Moderate	64 (10.5)/610	9.3 (7.2–11.9)	7.82 (5.36–11.39)	<0.001	6.14 (4.26–8.86)	<0.001	5.17 (3.35–7.96)	<0.001
High	36 (5.9)/610	10.1 (7.2–13.9)	8.59 (5.37–13.75)	<0.001	8.42 (5.34–13.29)	<0.001	5.98 (3.62–9.87)	<0.001

^a^Model 1: adjusted for sociodemographic information (i.e., age, sex, BMI, marital status, working status, educational level, smoking status, alcohol drinking status, reimbursement scheme, and living distance from PD center).

^b^Model 2: adjusted for model 1 plus clinical characteristics and PD practices (i.e., year of PD initiation, PD centers, eGFR at PD initiation, urgent-start PD, PD modality, RKF at PD initiation, etiology of ESKD, and non-age-adjusted Charlson comorbidity index.

^c^Model 3 (full model): adjusted for model 2 plus laboratory profiles (i.e., creatinine, urea nitrogen, serum albumin, hemoglobin, ferritin, transferrin saturation, sodium, potassium, bicarbonate, calcium, phosphorus, intact parathyroid hormone, neutrophil-to-lymphocyte ratio, platelet-to-lymphocyte ratio, and alkaline phosphatase).

^d^Based on the cox proportional hazards model with shared frailty correction analysis.

BMI, body mass index; CI, confidence interval; CoNS, Coagulase-negative Staphylococci; eGFR, estimated glomerular filtration rate; ESI, exit-site infection; ESKD, end-stage kidney disease; HR, hazard ratio; PD, peritoneal dialysis; THOR-PD, Thai renal outcomes research-peritoneal dialysis.

### Further analysis

With respect to the secondary analysis (Table 2 and Supplementary Table S5), all additional risk estimates according to causative organism of ESI (Gram-positive or Gram-negative), common pathologic organism of ESI (*S. aureus* or CoNS), and severity strata of causative organism of ESI (low, moderate, high-risk of mortality or catheter removal) were statistically significant: full adjusted HRs ranged from 5.17 to 5.98, with all *p* < 0.001.

Regarding *a priori* sensitivity analyses, the effect estimates from *post hoc* analyses without shared frailty correction and excluding individuals with culture-negative ESIs were all robust and similar to the main findings (Supplementary Tables S6, S7). Nevertheless, the association between ESI and subsequent risk of PD-associated peritonitis could not be fully addressed for residual confounders, as indicated by the *E*-value corresponding to the lower limit of 95% CIs for all full model effect estimates (*E*-values ranged and 95% CIs lower limit ranged from 3.64 to 35.79 and 2.62 to 13.00, respectively, Supplementary Table S8 and Supplementary Figure S1).

### Ancillary analysis with meta-analysis

Based on systematic searching, 3,844 and 5 records were identified through electronic databases and grey literature, respectively (Supplementary Figure S2). Following systematic screening, 77 articles were then reviewed against study selection criteria. Of these, 75 articles were excluded (details of the reasons for exclusions are provided in Supplementary Table S9). Finally, 2 cohort studies (12, 14) conducted in Canada (a secondary analysis of a randomized clinical trial and a retrospective cohort) were eligible for ancillary analysis and meta-analysis, incorporating evidence into this study. Regarding the study quality assessment, all included studies were rated as high quality (details are provided in Supplementary Table S10). Table 3 summarizes key characteristics of the cohort included in the ancillary analysis with meta-analysis.

**Table 3:** Key characteristics of cohort studies included in the ancillary analysis with meta-analysis.

Study characteristics	van Diepen et al, 2012 (MP3 cohort) (14)	Lloyd et al, 2013 (PD-MAN cohort) (12)	This study, 2026 (THOR-PD cohort)
Total sample size	203	962	610
Country (design)	Canada (secondary analysis of a prospective, multicenter, randomized clinical trial)	Canada (retrospective cohort)	Thailand (retrospective cohort)
Study population	Incident and prevalent cases of adult PD patients aged >18 years who were followed as part of the clinical trial that compared 2 PD catheter exit-site ointments—the Mupirocin Versus Polysporin Triple (MP3) study	Incident cases of adult PD patients from Manitoba province, between January 1, 2000, and December 31, 2009, based on a local administrative database	Incident cases of adult PD patients from 3 large PD centers in Chiang Mai province, between January 1, 2006, and September 30, 2022, based on routine longitudinal merged patient-level data
Received antibiotics at PD catheter insertion	Not specified	Yes	Yes
Median follow-up, years	1.5	1.6	1.5
Total patient-years of follow-up	229.7	1984	1107.7
Age, mean (SD), years	60.5 (14.4)	55.6 (15.6)^a^	59.3 (10.7)
Male sex, No. (%)	131 (64.5)	477 (51.2)^a^	346 (56.7)
BMI, mean (SD), kg/m^2^	Not reported	27.3 (6.3)^a^	22. 0 (3.4)
CAPD, No. (%)	96 (47.3)	325 (34.9)^a^	543 (89.0)
Diabetes, No. (%)	88 (43.3)	437 (46.9)^a^	256 (42.0)
Exposure	Based on the ISPD definition, 34 patients experienced 44 episodes of ESI^b^	Based on the ISPD definition, 440 patients with 962 episodes of ESI^b^	Based on the ISPD definition, 305 patients experienced the first episode of ESI^b^
Included episodes of exit-site infection with culture negative [No. (%)]	Yes [5 episodes (11.4)]	Yes [49 episodes (5.1)]	Yes [29 episodes (9.5)]
Adjustment variables	Age, sex, and diabetes	Matched pair based on time-to-ESI (time-matched)^c^ with adjusted variables (sex, diabetes, Aboriginal peoples)	Matched pair with age, sex, and time-to-index date and hierarchically adjusted based on patients’ sociodemographic, clinical characteristics and PD practices, and laboratory profiles (see details in methods)
Outcomes	Based on the ISPD diagnostic criteria, 57 individuals experienced 87 peritonitis episodes^d^	Based on the ISPD diagnostic criteria, overall, 1,228 peritonitis episodes^d^	Based on the ISPD diagnostic criteria, 414 patients experienced with PD-associated peritonitis^d^
Quality assessment (summary NOS score)	High quality (8)^e^	High quality (8)^e^	High quality (9)^e^

^a^Based on the sample cohort of individuals with and without exit-site infection (*n* = 931).

^b^Defined as purulent drainage from the exit-site with or without erythema.

^c^Patients could have multiple episodes of exit-site infection and could be matched more than once.

^d^Defined as individuals who met at least 2 criteria of the following: (i) clinical features consistent with peritonitis (i.e., abdominal pain and/or cloudy dialysis effluent); (ii) dialysis effluent white blood cell count >100 cells/μL (>50% polymorphonuclear leukocytes); and (iii) positive dialysate effluent culture.

^e^See details in Supplementary Table S10.

ESI, exit-site infection; ISPD, International society for peritoneal dialysis; NOS, Newcastle-Ottawa Scale; PD, peritoneal dialysis.

**Figure 3:**
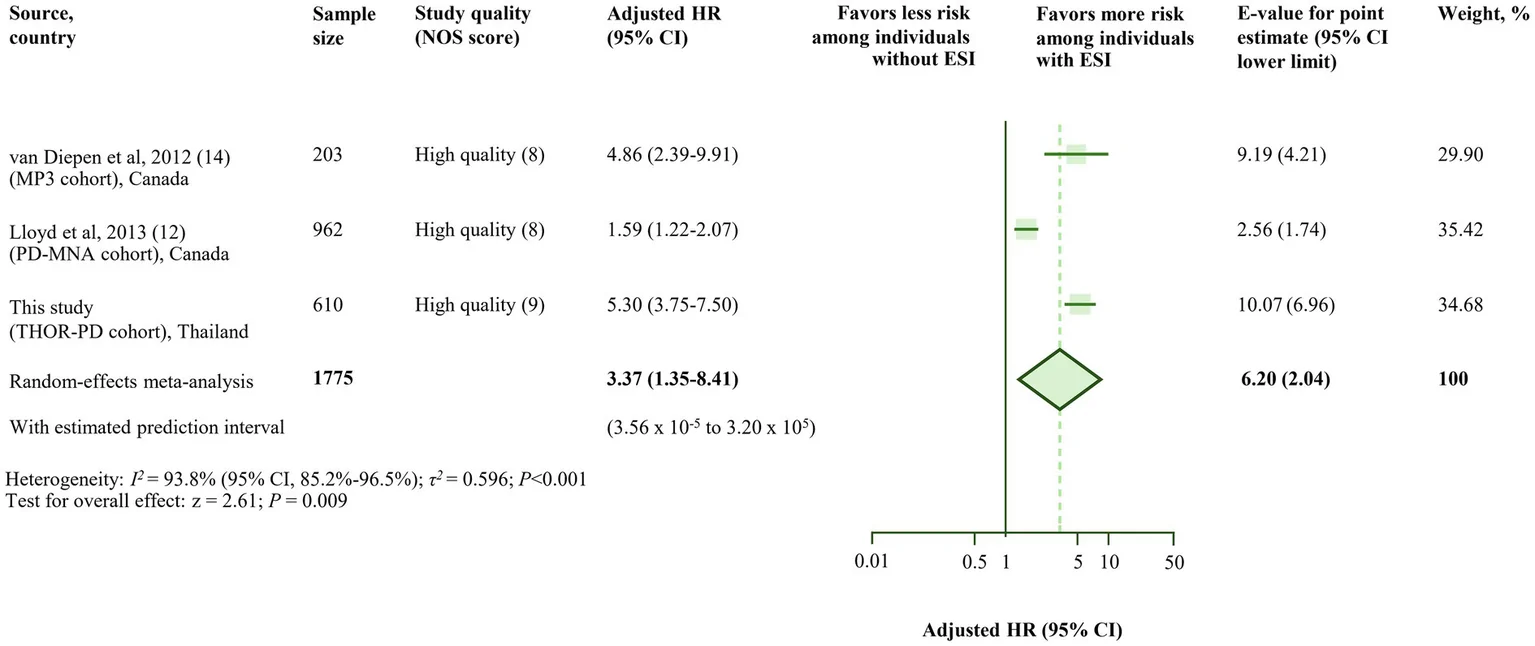
Ancillary analysis with meta-analytic results of ESI and the subsequent risk of PD-associated peritonitis across the 3 cohort studies. CI, confidence interval; ESI, exit-site infection; HR, hazard ratio; NOS, Newcastle-Ottawa Scale; PD, peritoneal dialysis.

With respect to random-effects models (Figure 3 and Supplementary Table S11), individuals experienced with ESI were associated with an increased subsequent risk of PD-associated peritonitis [3 cohort studies (12, 14); *n* = 1775; adjusted HR, 3.37 (95% CI, 1.35–8.41); *p* = 0.009; with high heterogeneity (*I*^2^ = 93.8%, *τ*^2^ = 0.596)]. Given the limited data available, *a priori* subgroup analyses and meta-regression to explore the effect modifier on the meta-analytic estimates, using study and patient characteristics, were not possible. However, based on the “leave-one-out” approach, the main meta-analytic synthesis appeared to be sensitive and inconsistent, with no association observed when a study by van Diepen et al. (14) or this study cohort was omitted (Supplementary Table S12).

The evidence certainty of the risk of PD-associated peritonitis after ESI was downgraded to very low strength of evidence due to study limitations, inconsistency, and evidence uncertainty in terms of prediction intervals, with a predicted range from 3.56 × 10^−5^ to 3.20 × 10^5^, and the E-value of 6.20 (95% CI lower limit, 2.04) (Supplementary Table S11 and Figure 3). Details of the evidence assessment are summarized in Supplementary Table S13. With respect to contextualized approaches of evidence-based conclusions, individuals experienced with ESI were classified as having a harmful effect with increased risk of PD-associated peritonitis.

## Discussion

### Overview of findings

In a retrospective cohort of incident PD cases in Thailand, we found that individuals with a first episode of ESI had approximately a 5-fold increase in the subsequent risk of PD-associated peritonitis, regardless of the causative organism or severity strata, compared with individuals without any episode of ESI during the PD treatment follow-up. The landmark analysis also indicates that the risk of PD-associated peritonitis is increased at a peak of 18-fold, within 1 month following ESI, and gradually decreases to 2-fold over the designed 9–12 time periods. Regarding ancillary meta-analysis with very low evidence certainty, we found that incorporating all available evidence into this cohort analysis also supported the main findings. Given the nature of retrospective cohort analysis and the limited evidence available in PD facilities, these findings appear to be influenced by residual or unmeasured confounders in the association between PD-associated peritonitis following ESI.

### Comparison with the relevant evidence

Considering the existing evidence available and to overcome methodological limitations in the previous literature, we expanded the knowledge gap by accounting for key modifiable and non-modifiable risk factors for PD-associated peritonitis that had not been previously addressed (12–14). To investigate causality, we rigorously defined and framed the index incident cases of exposure (i.e., consider only the first episode of ESI) and non-exposure groups (i.e., consider only individuals without any episodes of ESI) using matched-pairs analysis and excluded cases that might interfere with the subsequent outcome of interest. Of these, to minimize subsequent effects of catheter-related infections, individuals with prior experience of PD catheter insertion-related infection or PD-associated peritonitis or tunnel infection, before or on the same day of the ESI, were excluded from our analysis. Based on the landmark analysis, the risk of PD-associated peritonitis is highest within 1 month after an ESI, with approximately 18-fold compared to individuals without any episode of ESI. These findings were supported by the previous secondary analysis of a randomized clinical trial (MP3 cohort), in which the observed risk is increased at a peak with 15 days (HR, 11.13; 95% CI, 4.94–25.07) and 1 month after ESI (HR, 6.32; 95% CI, 2.85–14.03) (14).

With respect to the available evidence, patient characteristics in our cohort were comparable to those in the existing studies by van Diepen et al. (14) and Lloyd et al. (12), with a mean age ranging from 59.3 to 60.5 years. Most cases were male (proportion of males ranged from 55.6 to 64.5%) and had a history of diabetes, ranging from 42.0 to 46.9%, representing the PD patients in daily practice. Considering differences in methodological design and analysis and substantial heterogeneity, our findings were consistent and supported by the 2 previous cohorts (12, 14). The overall HRs for developing PD-associated peritonitis following ESI across all 3 cohorts, including this study, ranged from 1.50 to 4.86. To provide more up-to-date evidence, we conducted an ancillary meta-analytic synthesis incorporating effect estimates from all 3 available cohorts, yielding a summary HR of 3.37 for PD-associated peritonitis.

Overall, our findings support an epidemiologic association concept that ESI may be associated with subsequent PD-associated peritonitis via translocation of microorganisms from the exit site into the peritoneal cavity (3, 7–11). Furthermore, we postulated that ESI may serve as a marker of increased susceptibility to infection, impaired host defense, suboptimal catheter care, or biofilm formation, rather than acting solely through direct extension of the causative organism.

Given the rigorous adjustment for covariates in an adequate sample, our findings appear to be sensitive to unmeasured or residual confounders. The E-value analysis suggests that an unmeasured confounder would need to have a relatively strong association with both ESI and PD-associated peritonitis, beyond the measured covariates, to explain the observed association fully. With respect to the potential unmeasured confounders, factors in relation to social determinants of health (SDOH) (e.g., environmental and community context, and economic stability), medical procedures and psychosocial issues, and other PD-related characteristics (e.g., patient preferences, choice of kidney replacement therapy, training system, variation in PD facilities, frailty and performance status, malnutrition-inflammatory complex syndrome, and self-care ability) that are directly and indirectly associated with the risk of PD-related infections are not fully acknowledged. We postulated that these multifactorial factors, at the patient level (and caregivers), mainly regarding sterile techniques, and at the PD center level, regarding environmental and infection control systems, may underpin the pathogenesis of PD-related infections, including ESIs and PD-associated peritonitis.

### Strengths and limitations

In this retrospective cohort of patient-level data, we performed a rigorous logical analysis of exposure (individuals experienced with the first episode of ESI) with corresponding matched-pair non-exposure (individuals without any episode of ESI), and subsequent risk of outcome of interest (PD-associated peritonitis) based on the contemporary definitions and treatment recommendations of the ISPD guidelines (9, 19). From a methodological viewpoint, we matched pairs of individuals at risk 1:1 and minimized confounding by accounting for hierarchical covariates to address knowledge gaps in the previous literature. We also performed a landmark analysis to account for immortal-time bias and to provide insight for clinical interpretation. Moreover, to draw evidence-based conclusions, an ancillary analysis using systematic review and meta-analysis was also supplemented to the main analysis, incorporating all available evidence into this cohort study.

Nevertheless, several pertinent limitations must be acknowledged. First, despite controlling multiple relevant covariates that address SDOH through sociodemographic gradients, differences in clinical characteristics and PD practices, and key laboratory profiles, unmeasured confounders, as estimated by E-values in all analyses, should be acknowledged. Several modifiable and non-modifiable risk factors for the development of PD-associated peritonitis reported in the literature were not available in this cohort, for instance, nutritional status and performance in physical and mental function. However, we considered several proxy covariates instead, such as the Charlson comorbidity index, serum albumin, ferritin level, and systemic immune-inflammation indices (i.e., neutrophil-to-lymphocyte ratio and platelet-to-lymphocyte ratio), to address this concern. Moreover, the risk estimates for PD-associated peritonitis after ESI were robust across a set of sensitivity analyses.

Second, although we restrict definitions of exposure and non-exposure to investigate the subsequent risk of the outcome, the causality relationship cannot be fully established due to the nature of the retrospective cohort design. Indeed, we considered only individuals with experience of the first episode of ESI and matched and analyzed them only once with counterpart individuals without any episode of ESI. Meanwhile, we also excluded individuals who had experienced PD-associated peritonitis or tunnel infection before or on the same day of ESI from our analysis. As a result, our analysis framework may not reflect all PD-related infection scenarios, including multiple PD-related infections such as repeated ESI and peritonitis, in daily practice.

Third, although a landmark analysis reaffirmed the concepts of designed timeframe relationships, the risk estimates gradually decreased over time. Unfortunately, details of the specific causative organisms of ESI and their subsequent causative organisms in PD-associated peritonitis were not addressed in this analysis, as they are outside the scope of this cohort study. Although we observed the consistent biologically plausible relationship between ESI and the subsequent risk of PD-associated peritonitis across all secondary analyses that accounted for the causative organism and its severity strata, our findings do not establish a direct microbiological transmission pathway. In fact, our findings should therefore be interpreted as demonstrating an epidemiologic association rather than establishing a causal microbiological pathway.

Fourth, this sample of the PD population was based solely on a particular policy-driven implementation, the “PD First” policy in Thailand, resulting in most of the sample representing CAPD patients (89.0%). Despite differences in PD centers being addressed in all analysis models, other variations in relation to PD center characteristics and practices over time should be considered, for instance, experiences of PD staff and practice patterns, and volume of uptake of PD cases, which may profoundly affect the quality of PD care and rates of PD-related infections in each PD center. Considering variation across PD facilities, the generalizability of our findings to other differences among healthcare providers and policies should be exercised with caution.

Finally, although an ancillary meta-analysis supported the association between ESI and the subsequent risk of PD-associated peritonitis, several preplanned meta-analytical syntheses to explore effect modifiers and the duration-response relationship could not be established due to limited available evidence. Of these, information was limited to adult PD populations and to only 2 countries (Canada and Thailand). Given the summary effect estimate, the sensitivity analysis, prediction intervals, and *E*-values, the uncertainty of the findings and potential residual confounders remain. As such, we downgraded the evidence and rated its strength as very low. Taken together, our findings must be interpreted in the context of the evidence’s certainty and the study’s limitations.

### Considerations for public infectious control and prevention, and future research

Collectively, by contextualizing evidence from the matched-pairs cohort and an ancillary meta-analysis, our findings reaffirmed the association between ESI and the subsequent risk of PD-associated peritonitis, particularly within 1 month of the infection. Theoretically, PD retraining, including primary caregivers, and intensive monitoring should be promptly implemented for individuals with ESI, particularly within 1 month of the infection. Unfortunately, as of today, few randomized clinical trials with relatively small samples have found no preferred option for exit-site care dressings to prevent catheter-related infections (9, 10, 19). As such, we underscore the need for effective, innovative pharmacological and non-pharmacological strategies, including continuous quality improvement studies, to prevent PD-related infections across diverse PD populations and facilities. Validated protocol components for interventions and PD program education following an ESI episode are also warranted to minimize individuals at risk of PD-associated peritonitis and promote patient health and a sustainable PD program.

From an epidemiological perspective, future studies with larger cohorts and diverse PD populations, more sophisticated studies using repeated time-to-event analysis for multiple exposures and outcomes, as well as analyses based on self-controlled designs, may help further clarify the temporal relationship between ESI and PD-associated peritonitis. Moreover, further studies that address duration-response to specific causative organisms, including *in vitro* and *in vivo* transcription models and host defense testing of biological pathways to develop PD-associated peritonitis after ESI, are also warranted.

## Conclusion

In this retrospective cohort, individuals with ESI had a substantial increase in the risk of PD-associated peritonitis in the subsequent period compared with those without ESI. Notably, based on landmark analysis, affected individuals are at elevated peak risk of developing PD-associated peritonitis within 1 month after experiencing ESI and gradually decrease over time. The findings remain robust across a set of secondary analyses according to the causative organism of ESI and sensitivity analyses. Given very low evidence certainty, an ancillary meta-analytical synthesis of all available evidence also supported these findings. Innovative strategies targeting protocols to prevent PD-related infections, including prompt interventions following an ESI episode, are warranted to minimize the risk of PD-associated peritonitis among individuals at risk and to promote patient health and sustainable PD programs.

## Data Availability

The raw data supporting the conclusions of this article will be made available by the authors, without undue reservation.
