# Supplementary material for: Exit-site infection and subsequent incident risk of peritoneal dialysis-associated peritonitis: a retrospective cohort study with meta-analysis
Source: Front Public Health. 2026 Aug 12;14:1893696. doi: 10.3389/fpubh.2026.1893696 (PMC13506861; doi:10.3389/fpubh.2026.1893696)
Supplement: Supplementary file 1 [file Supplementary_file_1.pdf]

Supplementary Online

Exit-Site Infection and Subsequent Incident Risk of Peritoneal Dialysis-Associated Peritonitis: A Retrospective Cohort Study with Meta-Analysis

Surapon Nochaiwong, Chidchanok Ruengorn, Kajohnsak Noppakun, Nuttaya Wachiraphansakul, Thanawat Vongchaiudomchoke, Tanun Ngamvichchukorn, Kednapa Thavorn, Manish M. Sood, Greg A. Knoll, Apichat Tantraworasin

Supplementary Online Content

**Table S1**	Missing data of covariates in the study cohort	S3
**Table S2**	Risk category of organism-specific peritonitis according to severity defined by mortality and catheter removal rate	S4
**Table S3**	Crude rates of PD-associated peritonitis after the first episode of exit-site infection	S5
**Table S4**	Multicollinearity diagnostics	S6
**Table S5**	Unadjusted risk of PD-associated peritonitis among individuals with exit-site infection in the matched-pair THOR-PD cohort	S7
**Table S6**	Sensitivity analysis: Cox proportional hazards model without shared frailty correction analysis	S8
**Table S7**	Sensitivity analysis: excluding individuals who experienced exit-site infection with culture-negative	S9
**Table S8**	Sensitivity analysis: E-value	S10
**Table S9**	Excluded studies	S11
**Table S10**	Study quality assessment: the Newcastle-Ottawa Scale for cohort studies	S16
**Table S11**	Summary of findings of ancillary analysis with meta-analysis	S17
**Table S12**	Sensitivity analysis: post-hoc analysis using the "leave-one-out" approach	S18
**Table S13**	Quality of evidence synthesis and GRADE evidence profile	S19
**Figure S1**	Estimation of E-Value	S20
**Figure S2**	Flowchart of study selection for systematic review and meta-analysis	S27
**Appendix S1**	Methods of ancillary analysis with systematic review and meta-analysis	S28
**eReferences**		S35

Table S1 Missing data of covariates in the study cohort

Variables	Missing	Total sample	Missing, %
Age (years)	0	610	0.00
Sex	0	610	0.00
Time-to-index date (time-matched)	0	610	0.00
BMI (kg/m^2)	0	610	0.00
Marital status	0	610	0.00
Woking status	0	610	0.00
Educational level	0	610	0.00
Smoking status	0	610	0.00
Alcohol drinking status	0	610	0.00
Reimbursement scheme	0	610	0.00
Living distance from PD center	0	610	0.00
Year of PD initiation	0	610	0.00
eGFR at PD initiation (mL/min/1.73 m^2)	0	610	0.00
Urgent-start PD	0	610	0.00
PD modality	0	610	0.00
Residual kidney function at PD initiation	0	610	0.00
Etiology of ESKD	0	610	0.00
Charlson comorbidity index (non-age adjusted)	0	610	0.00
Diabetes mellitus	0	610	0.00
Coronary artery disease	0	610	0.00
Chronic heart failure	0	610	0.00
Cerebrovascular disease	0	610	0.00
Serum creatinine (mg/dL)	0	610	0.00
Serum urea nitrogen (mg/dL)	0	610	0.00
Serum albumin (g/dL)	0	610	0.00
Hemoglobin (g/dL)	0	610	0.00
Ferritin (ng/mL)	4	610	0.66
Transferrin saturation, %	12	610	1.97
Sodium (mEq/L)	0	610	0.00
Potassium (mEq/L)	0	610	0.00
Bicarbonate (mEq/L)	0	610	0.00
Calcium (mg/dL)	0	610	0.00
Phosphorus (mg/dL)	0	610	0.00
Intact parathyroid hormone (pg/mL)	13	610	2.13
Neutrophil-to-lymphocyte ratio	0	610	0.00
Platelet-to-lymphocyte ratio	0	610	0.00
Alkaline phosphatase (U/L)	12	610	1.97

Abbreviations: BMI, body mass index; eGFR, estimated glomerular filtration; ESKD, end-stage kidney disease; PD, peritoneal dialysis.

Table S2 Risk category of organism-specific peritonitis according to severity strata defined by mortality and catheter removal rate

Severity Strata	Mortality Rate	Catheter Removal Rate	Microbiology
Low	<3%	<10%	• Streptococcal<br>• CoNS<br>• *Staphylococcus aureus* (excluding MRSA)<br>• Other gram-positive<br>• Culture-negative
Moderate	3-5%	10-20%	• *Enterococcus*<br>• *Corynebacterium*<br>• Non-*Pseudomonas* gram-negative
High	>5%	>20%	• MRSA<br>• *Pseudomonas*<br>• Polymicrobial<br>• Mycobacterium<br>• Fungal

Abbreviations: CoNS, Coagulase-negative *Staphylococci*; MRSA, methicillin-resistant *S. aureus*; PD, peritoneal dialysis.

Table S3 Crude rates of PD-associated peritonitis after the first episode of exit-site infection

Outcomes	Overall	Individuals without any episode of exit-site infection	Individuals with exit-site infection
• No. of PD-associated peritonitis (%)/total sample	414 (67.9)/610	286 (93.8)/305	128 (42.0)/305
• Patient-years at risk of PD-associated peritonitis[a]	1107.7	824.1	283.6
• Median time survival of PD-associated peritonitis, year (95% CI)[a]	1.5 (1.2 – 1.8)	3.9 (3.3 – NA)	0.5 (0.4 – 0.7)
• Incidence rate of PD-associated peritonitis per 100 patient-years (95% CI)[a]	3.1 (2.8 – 3.4)	1.3 (1.1 – 1.5)	8.4 (7.5 – 9.4)
• Absolute risk difference (crude %)		59.4 (53.3 – 65.5)	
• Patient-years of follow-up[b]	2540.9	1378.5	1162.4
• Median time survival, year (95% CI)[b]	8.0 (6.8 – 10.1)	8.6 (7.5 – 10.8)	6.5 (5.2 – NA)

[a]Time at risk was defined as the index date (the date of the first episode of exit-site infection) to the occurrence of PD-associated peritonitis, the end of study, or the censored date, whichever came first.
[b]Time at risk was defined as the PD initiation date to the end of the study or the censored date, whichever came first.
Abbreviations: CI, confidence interval; NA, not applicable; PD, peritoneal dialysis.

Table S4 Multicollinearity diagnostics

Variables	VIF[a]	Tolerance[b]
Age (years)	1.73	0.58
Sex	1.19	0.84
BMI (kg/m^2)	1.12	0.89
Marital status	1.31	0.76
Woking status	1.23	0.81
Educational level	1.29	0.78
Smoking status	1.20	0.84
Alcohol drinking status	1.24	0.81
Reimbursement scheme	1.61	0.62
Living distance from PD center	1.11	0.90
Year of PD initiation	1.15	0.87
PD center	1.41	0.71
eGFR at PD initiation	1.18	0.84
Urgent-start PD	1.13	0.88
PD modality	1.56	0.64
Residual kidney function at PD initiation	1.09	0.92
Etiology of ESKD	1.09	0.92
Charlson comorbidity index (non-age adjusted)	1.42	0.71
Serum creatinine (mg/dL)	1.49	0.67
Serum urea nitrogen (mg/dL)	1.57	0.64
Serum albumin (g/dL)	1.35	0.74
Hemoglobin (g/dL)	1.26	0.79
Ferritin (ng/mL)	1.51	0.66
Transferrin saturation, %	1.12	0.90
Sodium (mEq/L)	1.19	0.84
Potassium (mEq/L)	1.24	0.80
Bicarbonate (mEq/L)	1.31	0.76
Calcium (mg/dL)	1.47	0.68
Phosphorus (mg/dL)	1.88	0.53
Intact parathyroid hormone (pg/mL)	1.87	0.53
Neutrophil-to-lymphocyte ratio	1.70	0.59
Platelet-to-lymphocyte ratio	1.34	0.75
Alkaline phosphatase (U/L)	1.77	0.56
**Mean VIF**	**1.37**	

Abbreviations: BMI, body mass index; eGFR, estimated glomerular filtration rate; ESKD, end-stage kidney disease; PD, peritoneal dialysis; VIF, variance inflation factor.

[a]VIF values <2.5 indicate no collinearity concern.

[b]Tolerance is the reciprocal of VIF; values of ≤0.40 are cause for concern.

Table S5 Unadjusted risk of PD-associated peritonitis among individuals with exit-site infection in the matched-pair THOR-PD cohort

Outcome analyses (reference: individuals without any episode of exit-site infection)	Unadjusted HR (95% CI)[a]	*P* value
**Main analysis**		
Overall risk of PD-associated peritonitis after the first episode of exit-site infection	7.74 (6.06 – 9.87)	<0.001
**Landmark analysis**		
Within 1 month after exit-site infection	20.05 (8.76 – 45.88)	<0.001
1 – 3 months after exit-site infection	18.03 (7.88 – 41.22)	<0.001
3 – 6 months after exit-site infection	4.88 (2.35 – 10.10)	<0.001
6 – 9 months after exit-site infection	3.21 (1.82 – 5.66)	<0.001
9 – 12 months after exit-site infection	2.06 (1.37 – 7.77)	<0.001
**Secondary analysis**		
According to causative organism of exit-site infection		
• Gram-positive	7.23 (5.52 – 9.47)	<0.001
• Gram-negative	8.31 (5.90 – 11.69)	<0.001
According to common pathologic organism of exit-site infection		
• *S. aureus*	7.08 (5.13 – 9.76)	<0.001
• CoNS	8.57 (5.78 – 12.71)	<0.001
According to severity strata of causative organism of exit-site infection		
• Low	7.42 (5.70 – 9.67)	<0.001
• Moderate	8.07 (5.58 – 11.69)	<0.001
• High	8.44 (5.33 – 13.38)	<0.001

[a]Based on the Cox proportional hazards model with shared frailty correction analysis.
Abbreviations: CI, confidence interval; CoNS, Coagulase-negative *Staphylococci*; HR, hazard ratio; PD, peritoneal dialysis; THOR-PD, Thai Renal Outcomes Research-Peritoneal Dialysis

Table S6 Sensitivity analysis: Cox proportional hazards model without shared frailty correction analysis

Outcome analyses (reference: individuals without exit-site infection)	HR (95% CI): Unadjusted	*P* value	HR (95% CI): Model 1[a]	*P* value	HR (95% CI): Model 2[b]	*P* value	HR (95% CI): Model 3[c]	*P* value
**Main analysis**								
Overall risk of PD-associated peritonitis after the first episode of exit-site infection	5.32 (4.28 – 6.61)	<0.001	5.58 (4.46 – 6.99)	<0.001	5.48 (4.33 – 6.95)	<0.001	5.00 (3.58 – 7.00)	<0.001
**Landmark analysis**								
Within 1 month after exit-site infection	19.24 (7.22 – 30.24)	<0.001	19.71 (7.40 – 31.42)	<0.001	18.81 (6.74 – 30.75)	<0.001	16.70 (5.00 – 32.46)	0.002
1 – 3 months after exit-site infection	13.53 (4.71 – 20.30)	<0.001	13.38 (4.59 – 19.16)	<0.001	11.07 (2.42 – 14.60)	0.004	11.02 (2.10 – 16.30)	0.032
3 – 6 months after exit-site infection	3.54 (1.74 – 7.20)	<0.001	3.46 (1.67 – 7.18)	0.001	4.19 (1.94 – 9.06)	<0.001	8.77 (2.66 – 28.97)	<0.001
6 – 9 months after exit-site infection	2.49 (1.43 – 6.32)	0.001	4.40 (1.38 – 6.20)	0.002	4.49 (1.37 – 6.52)	0.001	3.68 (1.70 – 6.06)	0.007
9 – 12 months after exit-site infection	2.98 (1.49 – 5.96)	0.002	2.74 (1.36 – 5.54)	0.005	3.22 (1.51 – 6.87)	0.003	2.55 (1.86 – 7.58)	0.042
**Secondary analysis**								
According to causative organism of exit-site infection								
• Gram-positive	4.96 (3.92 – 6.28)	<0.001	5.38 (4.21 – 6.87)	<0.001	5.33 (4.13 – 6.88)	<0.001	4.94 (3.48 – 7.01)	<0.001
• Gram-negative	6.31 (4.71 – 8.44)	<0.001	6.06 (4.49 – 8.18)	<0.001	5.97 (4.37 – 8.16)	<0.001	5.14 (3.45 – 7.64)	<0.001
According to common pathologic organism of exit-site infection								
• *S. aureus*	4.86 (3.70 – 6.39)	<0.001	5.55 (4.16 – 7.39)	<0.001	5.90 (4.37 – 7.96)	<0.001	5.27 (3.53 – 7.87)	<0.001
• CoNS	5.83 (4.22 – 8.07)	<0.001	5.94 (4.26 – 8.28)	<0.001	5.36 (3.79 – 7.56)	<0.001	5.08 (3.37 – 7.67)	<0.001
According to severity strata of causative organism of exit-site infection								
• Low	5.06 (4.01 – 6.38)	<0.001	5.41 (4.26 – 6.88)	<0.001	5.37 (4.17 – 6.91)	<0.001	4.89 (3.45 – 6.93)	<0.001
• Moderate	5.81 (4.26 – 7.91)	<0.001	5.82 (4.24 – 7.98)	<0.001	5.20 (3.74 – 7.23)	<0.001	4.95 (3.26 – 7.51)	<0.001
• High	6.16 (4.22 – 8.98)	<0.001	6.21 (4.20 – 9.18)	<0.001	6.90 (4.59 – 10.37)	<0.001	5.62 (3.48 – 9.05)	<0.001

[a]Model 1: adjusted for sociodemographic information (i.e., age, sex, BMI, marital status, working status, educational level, smoking status, alcohol drinking status, reimbursement scheme, and living distance from PD center).

[b]Model 2: adjusted for model 1 plus clinical characteristics and PD practices (i.e., year of PD initiation, PD centers, eGFR at PD initiation, urgent-start PD, PD modality, RKF at PD initiation, etiology of ESKD, and non-age-adjusted Charlson comorbidity index.

[c]Model 3 (full model): adjusted for model 2 plus laboratory profiles (i.e., creatinine, urea nitrogen, serum albumin, hemoglobin, ferritin, transferrin saturation, sodium, potassium, bicarbonate, calcium, phosphorus, intact parathyroid hormone, neutrophil-to-lymphocyte ratio, platelet-to-lymphocyte ratio, and alkaline phosphatase).

Abbreviations: BMI, body mass index; CI, confidence interval; CoNS, Coagulase-negative *Staphylococci*; eGFR, estimated glomerular filtration rate; ESKD, end-stage kidney disease; HR, hazard ratio; PD, peritoneal dialysis; THOR-PD, Thai Renal Outcomes Research-Peritoneal Dialysis.

Table S7 Sensitivity analysis: excluding individuals who experienced exit-site infection with culture-negative

Outcome analyses (reference: individuals without exit-site infection)	HR (95% CI): Unadjusted	*P* value	HR (95% CI): Model 1[a]	*P* value	HR (95% CI): Model 2[b]	*P* value	HR (95% CI): Model 3[c]	*P* value
**Main analysis**								
Overall risk of PD-associated peritonitis after the first episode of exit-site infection	7.68 (5.98 – 9.86)	<0.001	7.66 (5.94 – 9.87)	<0.001	6.57 (5.08 – 8.50)	<0.001	5.39 (3.77 – 7.70)	<0.001
**Landmark analysis**								
Within 1 month after exit-site infection	20.44 (8.84 – 47.27)	<0.001	36.78 (13.56 – 99.73)	<0.001	29.76 (9.45 – 93.67)	<0.001	17.70 (6.91 – 40.46)	<0.001
1 – 3 months after exit-site infection	16.48 (7.06 – 38.46)	<0.001	25.72 (9.55 – 69.27)	<0.001	7.33 (2.72 – 19.76)	<0.001	9.12 (2.87 – 16.74)	<0.001
3 – 6 months after exit-site infection	4.77 (2.19 – 10.39)	<0.001	4.72 (2.29 – 10.99)	<0.001	4.55 (1.97 – 10.52)	<0.001	6.17 (3.21 – 13.63)	<0.001
6 – 9 months after exit-site infection	3.46 (1.94 – 6.17)	<0.001	3.38 (1.85 – 6.16)	<0.001	3.67 (1.84 – 7.31)	<0.001	3.33 (1.10 – 10.04)	<0.001
9 – 12 months after exit-site infection	1.77 (1.19 – 7.39)	<0.001	1.55 (1.68 – 7.52)	<0.001	1.84 (1.35 – 6.06)	<0.001	1.91 (1.32 – 6.19)	<0.001
**Secondary analysis**								
According to causative organism of exit-site infection								
• Gram-positive	7.21 (5.50 – 9.45)	<0.001	7.44 (5.65 – 9.81)	<0.001	6.38 (4.85 – 8.41)	<0.001	5.30 (3.66 – 7.67)	<0.001
• Gram-negative	8.30 (5.89 – 11.69)	<0.001	7.78 (5.49 – 11.01)	<0.001	6.91 (4.89 – 9.75)	<0.001	5.45 (3.58 – 8.29)	<0.001
According to common pathologic organism of exit-site infection								
• *S. aureus*	7.09 (5.13 – 9.80)	<0.001	7.66 (5.49 – 10.68)	<0.001	6.95 (4.99 – 9.67)	<0.001	5.70 (3.71 – 8.75)	<0.001
• CoNS	8.59 (5.79 – 12.76)	<0.001	8.11 (5.42 – 12.11)	<0.001	6.58 (4.48 – 9.69)	<0.001	5.58 (3.59 – 8.68)	<0.001
According to severity strata of causative organism of exit-site infection								
• Low	7.27 (5.52 – 9.58)	<0.001	7.44 (5.63 – 9.84)	<0.001	6.41 (4.85 – 8.48)	<0.001	5.29 (3.65 – 7.68)	<0.001
• Moderate	8.10 (5.59 – 11.75)	<0.001	7.75 (5.32 – 11.28)	<0.001	6.04 (4.18 – 8.72)	<0.001	5.26 (3.38 – 8.16)	<0.001
• High	8.45 (5.33 – 13.40)	<0.001	8.53 (5.34 – 13.62)	<0.001	8.50 (5.37 – 13.43)	<0.001	6.09 (3.65 – 10.16)	<0.001

[a]Model 1: adjusted for sociodemographic information (i.e., age, sex, BMI, marital status, working status, educational level, smoking status, alcohol drinking status, reimbursement scheme, and living distance from PD center).
[b]Model 2: adjusted for model 1 plus clinical characteristics and PD practices (i.e., year of PD initiation, PD centers, eGFR at PD initiation, urgent-start PD, PD modality, RKF at PD initiation, etiology of ESKD, and non-age-adjusted Charlson comorbidity index.
[c]Model 3 (full model): adjusted for model 2 plus laboratory profiles (i.e., creatinine, urea nitrogen, serum albumin, hemoglobin, ferritin, transferrin saturation, sodium, potassium, bicarbonate, calcium, phosphorus, intact parathyroid hormone, neutrophil-to-lymphocyte ratio, platelet-to-lymphocyte ratio, and alkaline phosphatase).
Abbreviations: BMI, body mass index; CI, confidence interval; CoNS, Coagulase-negative *Staphylococci*; eGFR, estimated glomerular filtration rate; ESKD, end-stage kidney disease; HR, hazard ratio; PD, peritoneal dialysis; THOR-PD, Thai Renal Outcomes Research-Peritoneal Dialysis.
Note: all analyses were based on Cox proportional hazards model with shared frailty correction analysis.

Table S8 Sensitivity analysis: E-value

Outcome analyses (reference: individuals without exit-site infection)	HR (95% CI): Model 3[a]	*P* value	E-value for point estimates (95% CI lower limit)
**Main analysis**			
Overall risk of PD-associated peritonitis after the first episode of exit-site infection	5.30 (3.75 – 7.50)	<0.001	10.07 (6.96)
**Landmark analysis**			
Within 1 month after exit-site infection	18.15 (6.76 – 40.27)	<0.001	35.79 (13.00)
1 – 3 months after exit-site infection	10.44 (2.98 – 17.21)	<0.001	20.37 (5.41)
3 – 6 months after exit-site infection	6.79 (3.77 – 15.29)	<0.001	13.06 (7.00)
6 – 9 months after exit-site infection	4.19 (2.78 – 8.18)	<0.001	7.85 (5.00)
9 – 12 months after exit-site infection	2.11 (1.62 – 7.89)	<0.001	3.64 (2.62)
**Secondary analysis**			
According to causative organism of exit-site infection			
• Gram-positive	5.24 (3.65 – 7.52)	<0.001	9.95 (6.76)
• Gram-negative	5.35 (3.54 – 8.07)	<0.001	10.17 (6.54)
According to common pathologic organism of exit-site infection			
• *S. aureus*	5.65 (3.72 – 8.58)	<0.001	10.78 (6.90)
• CoNS	5.45 (3.54 – 8.40)	<0.001	10.38 (6.54)
According to severity strata of causative organism of exit-site infection			
• Low	5.21 (3.64 – 7.46)	<0.001	9.89 (6.74)
• Moderate	5.17 (3.35 – 7.96)	<0.001	9.81 (6.16)
• High	5.98 (3.62 – 9.87)	<0.001	11.44 (6.70)

[a]Model 3 (full model): adjusted for model 2 plus laboratory profiles (i.e., creatinine, urea nitrogen, serum albumin, hemoglobin, ferritin, transferrin saturation, sodium, potassium, bicarbonate, calcium, phosphorus, intact parathyroid hormone, neutrophil-to-lymphocyte ratio, platelet-to-lymphocyte ratio, and alkaline phosphatase).

Abbreviations: BMI, body mass index; CI, confidence interval; CoNS, Coagulase-negative *Staphylococci*; eGFR, estimated glomerular filtration rate; ESKD, end-stage kidney disease; HR, hazard ratio; PD, peritoneal dialysis; THOR-PD, Thai Renal Outcomes Research-Peritoneal Dialysis.

Note: all analyses were based on Cox proportional hazards model with shared frailty correction analysis.

Table S9 Excluded studies

List	Author (Year)	Title	Reason for Exclusion
1	Kim D, et al (1984)	Staph aureus peritonitis in patients on continuous ambulatory peritoneal dialysis. Trans Am Soc Artif Intern Organs.1984; 30 494-7.	Not relevant in the context of exit-site infection and subsequent risk of peritoneal dialysis-related peritonitis
2	Nolph KD, et al (1985)	A Randomized Multicenter Clinical Trial to Evaluate the Effects of An Ultraviolet Germicidal System on Peritonitis Rate in Continuous Ambulatory Peritoneal Dialysis. Perit Dial Int.1985; 5 (1): 19-24.	Not relevant in the context of exit-site infection and subsequent risk of peritoneal dialysis-related peritonitis
3	Piraino B, et al (1986)	The Influence of Peritoneal Catheter Exit-Site Infections on Peritonitis, Tunnel Infections, and Catheter Loss in Patients on Continuous Ambulatory Peritoneal Dialysis. Am J Kidney Dis.1986; 8 (6): 436-440.	Specific effect estimate (time-to-event, hazard ratios) of interest not reported
4	Piraino B, et al (1987)	A five-year study of the microbiologic results of exit site infections and peritonitis in continuous ambulatory peritoneal dialysis. Am J Kidney Dis.1987; 10 (4): 281-6.	Specific effect estimate (time-to-event, hazard ratios) of interest not reported
5	Abraham G, et al (1988)	Natural history of exit-site infection (ESI) in patients on continuous ambulatory peritoneal dialysis (CAPD). Perit Dial Int.1988; 8 (3): 211-216.	Specific effect estimate (time-to-event, hazard ratios) of interest not reported
6	Levy M, et al (1990)	Exit-site infection during continuous and cycling peritoneal dialysis in children. Perit Dial Int.1990; 10 (1): 31-5.	Specific effect estimate (time-to-event, hazard ratios) of interest not reported
7	Holley J, et al (1991)	Risk Factors for Tunnel Infections in Continuous Peritoneal Dialysis. Am J Kidney Dis.1991; 18 (3): 344-348.	Not relevant in the context of exit-site infection and subsequent risk of peritoneal dialysis-related peritonitis
8	Scalamogna A, et al (1991)	Exit-site and tunnel infections in continuous ambulatory peritoneal dialysis patients. Am J Kidney Dis.1991; 18 (6): 674-7.	Specific effect estimate (time-to-event, hazard ratios) of interest not reported
9	Lee GS, et al (1992)	Infection in continuous ambulatory peritoneal dialysis (CAPD): aetiology, complications and risk factors. Ann Acad Med Singap.1992; 21 (3): 354-60.	No outcomes of interest reported
10	Oxton LL, et al (1994)	Risk factors for peritoneal dialysis-related infections. Perit Dial Int.1994; 14 (2): 137-44.	Not relevant in the context of exit-site infection and subsequent risk of peritoneal dialysis-related peritonitis
11	Bunke M, et al (1995)	Pseudomonas peritonitis in peritoneal dialysis patients: the Network #9 Peritonitis Study. Am J Kidney Dis.1995; 25 (5): 769-74.	Specific effect estimate (time-to-event, hazard ratios) of interest not reported
12	Golper TA, et al (1996)	Risk factors for peritonitis in long-term peritoneal dialysis: the Network 9 peritonitis and catheter survival studies. Academic Subcommittee of the Steering Committee of the Network 9 Peritonitis and Catheter Survival Studies. Am J Kidney Dis.1996; 28 (3): 428-36.	Not relevant in the context of exit-site infection and subsequent risk of peritoneal dialysis-related peritonitis
13	Gupta B, et al (1996)	Peritonitis associated with exit site and tunnel infections. Am J Kidney Dis.1996; 28 (3): 415-9.	Not relevant in the context of exit-site infection and subsequent risk of peritoneal dialysis-related peritonitis
14	Paquay YC, et al (1996)	Long-term clinical experience with continuous ambulatory peritoneal dialysis: access-related problems. J Invest Surg.1996; 9 (2): 81-93.	Specific effect estimate (time-to-event, hazard ratios) of interest not reported
15	Caravaca F, et al (1998)	Risk factors for developing peritonitis caused by micro-organisms of enteral origin in peritoneal dialysis patients. Perit Dial Int.1998; 18 (1): 41-5.	Specific effect estimate (time-to-event, hazard ratios) of interest not reported
16	Crabtree JH, et al (1999)	Dialysis catheter infection related peritonitis: incidence and time dependent risk. ASAIO J.1999; 45 (6): 574-80.	Specific effect estimate (time-to-event, hazard ratios) of interest not reported
17	Szabo T, et al (1999)	Outcome of Pseudomonas aeruginosa exit-site and tunnel infections: a single center's experience. Adv Perit Dial.1999; 15 209-212.	No outcomes of interest reported

Table S9 Excluded studies (continued)

List	Author (Year)	Title	Reason for Exclusion
18	Furth SL, et al (2000)	Peritoneal dialysis catheter infections and peritonitis in children: a report of the North American Pediatric Renal Transplant Cooperative Study. Pediatr Nephrol.2000; 15 (3-4): 179-82.	Not relevant in the context of exit-site infection and subsequent risk of peritoneal dialysis-related peritonitis
19	Szeto CC, et al (2001)	Clinical course of peritonitis due to Pseudomonas species complicating peritoneal dialysis: a review of 104 cases. Kidney Int.2001; 59 (6): 2309-15.	Not relevant in the context of exit-site infection and subsequent risk of peritoneal dialysis-related peritonitis
20	Szeto CC, et al (2003)	Influence of climate on the incidence of peritoneal dialysis-related peritonitis. Perit Dial Int.2003; 23 (6): 580-6.	Specific effect estimate (time-to-event, hazard ratios) of interest not reported
21	Boehm M, et al (2005)	Risk factors for peritonitis in pediatric peritoneal dialysis: A single-center study. Pediatr Nephrol.2005; 20 (10): 1478-1483.	Specific effect estimate (time-to-event, hazard ratios) of interest not reported
22	Szeto CC, et al (2006)	Enterobacteriaceae peritonitis complicating peritoneal dialysis: a review of 210 consecutive cases. Kidney Int.2006; 69 (7): 1245-52.	Not relevant in the context of exit-site infection and subsequent risk of peritoneal dialysis-related peritonitis
23	Szeto CC, et al (2007)	Staphylococcus aureus peritonitis complicates peritoneal dialysis: review of 245 consecutive cases. Clin J Am Soc Nephrol.2007; 2 (2): 245-51.	Not relevant in the context of exit-site infection and subsequent risk of peritoneal dialysis-related peritonitis
24	Chiu MC, et al (2008)	Peritonitis and exit-site infection in pediatric automated peritoneal dialysis. Perit Dial Int.2008; 28 Suppl 3 S179-82.	Specific effect estimate (time-to-event, hazard ratios) of interest not reported
25	Szeto CC, et al (2009)	Recurrent and relapsing peritonitis: causative organisms and response to treatment. Am J Kidney Dis.2009; 54 (4): 702-710.	Not relevant in the context of exit-site infection and subsequent risk of peritoneal dialysis-related peritonitis
26	Cleper R, et al (2010)	Peritonitis in a pediatric dialysis unit: Local profile and implications. Isr Med Assoc J.2010; 12 (6): 348-352.	Not relevant in the context of exit-site infection and subsequent risk of peritoneal dialysis-related peritonitis
27	Kofteridis DP, et al (2010)	Peritoneal dialysis-associated peritonitis: clinical features and predictors of outcome. Int J Infect Dis.2010; 14 (6): e489-93.	Not relevant in the context of exit-site infection and subsequent risk of peritoneal dialysis-related peritonitis
28	Lobo JV, et al (2010)	Predictor factors of peritoneal dialysis-related peritonitis. J Bras Nefrol.2010; 32 (2): 156-164.	Specific effect estimate (time-to-event, hazard ratios) of interest not reported
29	Raaijmakers R, et al (2010)	Peritonitis in children on peritoneal dialysis in Cape Town, South Africa: Epidemiology and risks. Pediatr Nephrol.2010; 25 (10): 2149-2157.	Not relevant in the context of exit-site infection and subsequent risk of peritoneal dialysis-related peritonitis
30	Al-Hwiesh AK, et al (2013)	Prevention of peritoneal dialysis catheter infections in Saudi peritoneal dialysis patients: the emergence of high-level mupirocin resistance. Int J Artif Organs.2013; 36 (7): 473-483.	Not relevant in the context of exit-site infection and subsequent risk of peritoneal dialysis-related peritonitis
31	Abud ACF, et al (2015)	Peritonitis and catheter exit-site infection in patients on peritoneal dialysis at home. Rev Lat Am Enfermagem. 2015;23(5):902-9.	Not relevant in the context of exit-site infection and subsequent risk of peritoneal dialysis-related peritonitis
32	Ozisik L, et al (2015)	The changing trends of peritoneal dialysis related peritonitis and novel risk factors. Ren Fail.2015; 37 (6): 1027-1032.	Specific effect estimate (time-to-event, hazard ratios) of interest not reported
33	Forbes TA, et al (2016)	Topical honey in the management of pediatric peritoneal dialysis exit sites. Perit Dial Int.2016; 36 (6): 684-687.	Case series
34	Muthucumarana K, et al (2016)	The Relationship Between Presentation and the Time of Initial Administration of Antibiotics With Outcomes of Peritonitis in Peritoneal Dialysis Patients: The PROMPT Study. Kidney Int Rep.2016; 1 (2): 65-72.	Not relevant in the context of exit-site infection and subsequent risk of peritoneal dialysis-related peritonitis
35	Sethna CB, et al (2016)	Risk factors for and outcomes of catheter-associated peritonitis in children: The SCOPE collaborative. Clin J Am Soc Nephrol.2016; 11 (9): 1590-1596.	Not relevant in the context of exit-site infection and subsequent risk of peritoneal dialysis-related peritonitis

Table S9 Excluded studies (continued)

List	Author (Year)	Title	Reason for Exclusion
36	Wu H, et al (2016)	Risk Factors for Early-Onset Peritonitis in Southern Chinese Peritoneal Dialysis Patients. Perit Dial Int.2016; 36 (6): 640-646.	Specific effect estimate (time-to-event, hazard ratios) of interest not reported
37	Htay H, et al (2017)	Comparison of Topical Chlorhexidine and Mupirocin for the Prevention of Exit-Site Infection in Incident Peritoneal Dialysis Patients. Perit Dial Int.2017; 37 (3): 266-272.	No outcomes of interest reported
38	Ong LM, et al (2017)	Risk of peritoneal dialysis-related peritonitis in a multi-racial asian population. Perit Dial Int.2017; 37 (1): 35-43.	Specific effect estimate (time-to-event, hazard ratios) of interest not reported
39	Wang LY, et al (2017)	Risk Factors for Early-onset Peritoneal Dialysis-associated Peritonitis. Chinese General Practice.2017; 20 (4): 454-458.	Specific effect estimate (time-to-event, hazard ratios) of interest not reported
40	Iida H, et al (2018)	Association between keeping home records of catheter exit-site and incidence of peritoneal dialysis-related infections. Int Urol Nephrol.2018; 50 (4): 763-769.	Not relevant in the context of exit-site infection and subsequent risk of peritoneal dialysis-related peritonitis
41	Swartz SJ, et al (2018)	Exit site and tunnel infections in children on chronic peritoneal dialysis: findings from the Standardizing Care to Improve Outcomes in Pediatric End Stage Renal Disease (SCOPE) Collaborative. Pediatr Nephrol.2018; 33 (6): 1029-1035.	No outcomes of interest reported
42	Beckwith H, et al (2019)	Repeat peritoneal dialysis exit-site infection: Definition and outcomes. Perit Dial Int.2019; 39 (4): 344-349.	Specific effect estimate (time-to-event, hazard ratios) of interest not reported
43	Chen HL, et al (2019)	Risk factors associated with outcomes of peritoneal dialysis in Taiwan: An analysis using a competing risk model. Medicine (Baltimore).2019;98(6):e14385.	Not relevant in the context of exit-site infection and subsequent risk of peritoneal dialysis-related peritonitis
44	Keswani M, et al (2019)	Risk factors for early onset peritonitis: the SCOPE collaborative. Pediatr Nephrol.2019; 34 (8): 1387-1394.	Not relevant in the context of exit-site infection and subsequent risk of peritoneal dialysis-related peritonitis
45	Milan Manani S, et al (2019)	Catheter-related infections in peritoneal dialysis: comparison of a single center results and the literature data. J Nephrol.2019; 32 (5): 837-841.	Not relevant in the context of exit-site infection and subsequent risk of peritoneal dialysis-related peritonitis
46	Sevinc M, et al (2019)	Comparison of Exit-Site Infection Frequency in Continuous Ambulatory Peritoneal Dialysis and Automated Peritoneal Dialysis Patients: A Single-Center Experience. Sisli Etfal Hastan Tip Bul.2019; 53 (4): 385-388.	Specific effect estimate (time-to-event, hazard ratios) of interest not reported
47	Silva F, et al (2019)	Exit-Site Infections in Peritoneal Dialysis: Predictive Factors for Adverse Outcomes. Bulletin de la Dialyse a Domicile.2019; 2 (3): 127-134.	No outcomes of interest reported
48	Abdul Rashid A, et al (2021)	Catheter-related infections and microbiological characteristics in coiled versus straight peritoneal dialysis catheters in Malaysia. Indian J Nephrol.2021; 31 (6): 511-515.	Cross-sectional studies
49	Rigo M, et al (2021)	Clinical utility of a traditional score system for the evaluation of the peritoneal dialysis exit-site infection in a national multicentric cohort study. Perit Dial Int.2021; 41 (3): 292-297.	Specific effect estimate (time-to-event, hazard ratios) of interest not reported
50	Nadarajah L, et al (2022)	Persistent colonization of exit site is associated with modality failure in peritoneal dialysis. Perit Dial Int.2022; 42 (1): 96-99.	No outcomes of interest reported
51	Yabe H, et al (2022)	Effects of cognitive impairment and assisted peritoneal dialysis on exit-site infection in older patients. Clin Exp Nephrol.2022; 26 (6): 593-600.	Not relevant in the context of exit-site infection and subsequent risk of peritoneal dialysis-related peritonitis
52	Ferreira AC, et al (2023)	Peritoneal Dialysis Exit-Site Care Protocols in Portugal and Its Association with Catheter-Related Infections. Blood Purifi.2023; 52 (4): 366-372.	Not relevant in the context of exit-site infection and subsequent risk of peritoneal dialysis-related peritonitis

Table S9 Excluded studies (continued)

List	Author (Year)	Title	Reason for Exclusion
53	Hayat A, et al (2023)	Associations, microbiology and outcomes of pre-training peritoneal dialysis-related peritonitis. Perit Dial Int.2023; 43 (2): 173-181.	Specific effect estimate (time-to-event, hazard ratios) of interest not reported
54	Kamath N, et al (2023)	Pediatric peritoneal dialysis training program and its relationship to peritonitis: a study of the International Pediatric Peritoneal Dialysis Network. Pediatr Nephrol.2023; 38 (12): 4111-4118.	Not relevant in the context of exit-site infection and subsequent risk of peritoneal dialysis-related peritonitis
55	Leung WY, et al (2023)	Retraining programme for older patients undergoing peritoneal dialysis: A randomised controlled trial. J Ren Care.2023; 49 (4): 253-263.	Not relevant in the context of exit-site infection and subsequent risk of peritoneal dialysis-related peritonitis
56	Young EW, et al (2023)	Peritoneal Dialysis-Associated Peritonitis Trends Using Medicare Claims Data, 2013-2017. Am J Kidney Dis.2023; 81 (2): 179-189.	Not relevant in the context of exit-site infection and subsequent risk of peritoneal dialysis-related peritonitis
57	Ito Y, et al (2024)	Risk factors of peritoneal dialysis-related peritonitis in the Japan Peritoneal Dialysis Outcomes and Practice Patterns Study (PDOPPS). Clin Kidney J.2024; 17 (7): sfae202.	Not relevant in the context of exit-site infection and subsequent risk of peritoneal dialysis-related peritonitis
58	Keswani M, et al (2024)	Interobserver agreement of peritoneal dialysis exit site scoring: Results from the Standardizing Care to Improve Outcomes in Pediatric End Stage Kidney Disease (SCOPE) collaborative. Perit Dial Int.2024; 44 (5): 390-396.	Not relevant in the context of exit-site infection and subsequent risk of peritoneal dialysis-related peritonitis
59	Liu D, et al (2024)	Microbiological characteristics and treatment outcome analysis of exit-site infection in peritoneal dialysis patients. Chinese Journal of Nephrology.2024; 40 (10): 798-803.	No outcomes of interest reported
60	Nakayama T, et al (2024)	Efficacy of sucrose and povidone-iodine mixtures in peritoneal dialysis catheter exit-site care. BMC Nephrol.2024; 25 (1): 151.	Not relevant in the context of exit-site infection and subsequent risk of peritoneal dialysis-related peritonitis
61	Nardelli L, et al (2024)	Tunnel ultrasound can guide the use of peritoneal dialysis catheter exit site relocation by external splicing and cuff removal in refractory tunnel infection. Int Urol Nephrol.2024; 56 (8): 2733-2741.	Case series
62	Tobijaszewska M, et al (2024)	A population-based study on the incidence and aetiology of infectious complications in peritoneal dialysis in South Sweden. Infect Dis (Lond).2024; 56 (3): 230-243.	Not relevant in the context of exit-site infection and subsequent risk of peritoneal dialysis-related peritonitis
63	Yaxley J, et al (2024)	Peritoneal dialysis catheterization with an upward tunnel and exit site: An observational study. J Vasc Access.2024.	Duplicate with Yaxley J, et al (2025)
64	Yooyen M, et al (2024)	Incidence and Factors Associated with Peritoneal Dialysis-Related Peritonitis in Children. J Med Assoc Thail.2024; 107 (5): 343-348.	Not relevant in the context of exit-site infection and subsequent risk of peritoneal dialysis-related peritonitis
65	Yuan D, et al (2024)	Analysis of risk factors for refractory peritonitis in patients undergoing peritoneal dialysis. Am J Transl Res.2024; 16 (8): 3832-3841.	Not relevant in the context of exit-site infection and subsequent risk of peritoneal dialysis-related peritonitis
66	Alfano G, et al (2025)	Screening for nasal carriage of Staphylococcus (S.) aureus reduces the risk of S. aureus peritonitis in patients on peritoneal dialysis (PD). Int Urol Nephrol. 2025;57(5):1639-1648.	Not relevant in the context of exit-site infection and subsequent risk of peritoneal dialysis-related peritonitis
67	Guo L, et al (2025)	Retrospective Analysis of Multi-Center Peritoneal Dialysis-Associated Peritonitis in Jiangsu Province: A Retrospective Analysis from 2016 to 2024. Journal of Natural Science, Biology and Medicine.2025; 16 (1): 201-210.	Specific effect estimate (time-to-event, hazard ratios) of interest not reported
68	Kim JH, et al (2025)	Characteristics of patients and facility of peritoneal dialysis in Korea: Results from the Peritoneal Dialysis Outcomes and Practice Patterns Study (PDOPPS) Korea. Perit Dial Int.2025; 45 (5): 281-290.	Not relevant in the context of exit-site infection and subsequent risk of peritoneal dialysis-related peritonitis
69	Ljungquist O, et al (2025)	Modifiable risk factors for peritoneal dialysis-related infections - a population-based cohort study on risk factors and outcomes in South Sweden. Front Nephrol. 2025;5:1583675.	Not relevant in the context of exit-site infection and subsequent risk of peritoneal dialysis-related peritonitis

Table S9 Excluded studies (continued)

List	Author (Year)	Title	Reason for Exclusion
70	Nakata J, et al (2025)	Examination of the Concordance Between Healthcare Professionals' and the Patient's Own Assessment of the Peritoneal Dialysis Catheter Exit Site. Ther Apher Dial.2025;29(6):898-904.	Not relevant in the context of exit-site infection and subsequent risk of peritoneal dialysis-related peritonitis
71	Soetendorp H, et al (2025)	Reduction of peritoneal dialysis associated infections using a novel exit-site care practice. Perit Dial Int.2025; 45 (2): 113-120.	Not relevant in the context of exit-site infection and subsequent risk of peritoneal dialysis-related peritonitis
72	Yaxley J, et al (2025)	Peritoneal dialysis catheterization with an upward tunnel and exit site: An observational study. J Vasc Access.2025;26(4):1318-1323.	Not relevant in the context of exit-site infection and subsequent risk of peritoneal dialysis-related peritonitis
73	Beckwith H, et al (2026)	Epidemiology of peritoneal dialysis exit site infections: Does sex play a role? Perit Dial Int.2026;46(3):261-264.	No outcomes of interest reported
74	Htay H, et al (2026)	Optimizing exit-site care for hospitalized peritoneal dialysis patients with weekly chlorhexidine-impregnated dressing. Perit Dial Int.2026;46(3):265-269.	Not relevant in the context of exit-site infection and subsequent risk of peritoneal dialysis-related peritonitis
75	Kuo PH, et al (2026)	Risk Factors for Peritonitis in Patients With Systemic Lupus Erythematosus Undergoing Peritoneal Dialysis. Int J Rheum Dis.2026;29(3):e70597.	No outcomes of interest reported

Table S10 Study quality assessment: the Newcastle-Ottawa Scale for cohort studies

First author (year)	Selection				Comparability		Outcomes			Total NOS
	Representativeness	Non-exposed: selection	Exposure: ascertainment	Outcomes not present at entry	Controls for: age, sex, serum albumin	Control for: additional factors[a]	Assessment	Follow-up long enough	Adequacy of follow-up	
van Diepen et al (2012)[1] (MP3 cohort)	*	*	*	*	…	*	*	*	*	8
Lloyd et al (2013)[2] (PD-MAN cohort)	*	*	*	*	…	*	*	*	*	8
This study (THOR-PD cohort)	*	*	*	*	*	*	*	*	*	9

[a]Study control for 3 additional factors as follows: age, sex, body mass index (obesity), socioeconomic, ethnicity/indigenous racial origin, living distantly from PD unit, pets, smoking, comorbid conditions (diabetes, coronary artery disease, chronic lung disease, hypertension, depression,), hypoalbuminemia, poor residual kidney function, hypokalemia, nasal *Staphylococcus aureus* carrier status, history of exit-site infection.

Abbreviations: NOS, Newcastle-Ottawa Scale (ranged 0-9); PD, peritoneal dialysis.

Table S11 Summary of findings of ancillary analysis with meta-analysis

**Outcomes (individuals with exit-site infection vs individuals without exit-site infection)**	**No. of included studies (sample size)**	**Random-effect model: adjusted HR (95% CI)**	***P*-value**	**E-values for**		**95% prediction interval**	**Heterogeneity**				**Strength of evidence**
				**Point estimate**	**CI limit**		***Q* statistic**	***P*-value**	**I^2 index (95% CI), %**	**τ^2**	
Overall risk of PD-associated peritonitis after exit-site infection	3 (1775)	3.37 (1.35 – 8.41)	0.009	6.20	2.04	3.56×10^{-5} to 3.20×10^{5}	32.49	<0.001	93.8 (85.2 – 96.5)	0.596	Low (harmful among individuals with exit-site infection)

Abbreviations: CI, confidence interval; HR, hazard ratio; PD, peritoneal dialysis.

Table S12 Sensitivity analysis: post-hoc analysis using the "leave-one-out" approach

First author (year)	HR (95% CI): Risk of PD-associated peritonitis after exit-site infection
**All studies**	
Omitted van Diepen et al (2012)[1] (MP3 cohort)	2.89 (0.89 – 9.39)
Omitted Lloyd et al (2013)[2] (PD-MAN cohort)	5.21 (3.82 – 7.12)
Omitted this study (THOR-PD cohort)	2.64 (0.89 – 7.86)

Abbreviations: CI, confidence interval; HR, hazard ratio; PD, peritoneal dialysis.

Table S13 Quality of evidence synthesis and GRADE evidence profile

Outcomes (exit-site infection vs non-exit-site infection)	No. of studies	Study design (sample size)	Quality assessment: required domains					Other issues	Finding and direction (magnitude) of Effect	Strength of evidence
			Study limitations	Directions	Consistency	Precision	Reporting bias			
Risk of PD-associated peritonitis	2 existing studies[1,2] and this cohort study (overall 3 studies)	Observational studies: 1 secondary analysis of prospective randomized clinical trials and 2 retrospective cohort (1775)	High	Direct	Consistency	Imprecision	Undetected	• Duration-response association could not be determined<br>• Present plausible confounding that would decrease the observed effect[a]	• Three observational studies with modest sample populations illustrated high study limitations and imprecise of evidence findings based on the sensitivity analyses and uncertainty of evidence findings (prediction interval).<br>• The summary pooled adjusted HR was 3.37 (95% CI, 1.35 – 8.41; P=0.009), with a high degree of heterogeneity (I^2, 93.8% [95% CI, 85.2 – 96.5]; τ^2=0.596).	Very low (harmful: increases risk among individuals experienced with exit-site infection compared to individuals without exit-site infection)

[a]On the basis of the E-value.

Abbreviations: CI, confidence interval; GRADE, Grading of Recommendations Assessment, Development and Evaluation; HR, hazard ratio; PD, peritoneal dialysis

Figure S1 Estimation of E-value

A. Overall risk of PD-associated peritonitis after the first episode of exit-site infection

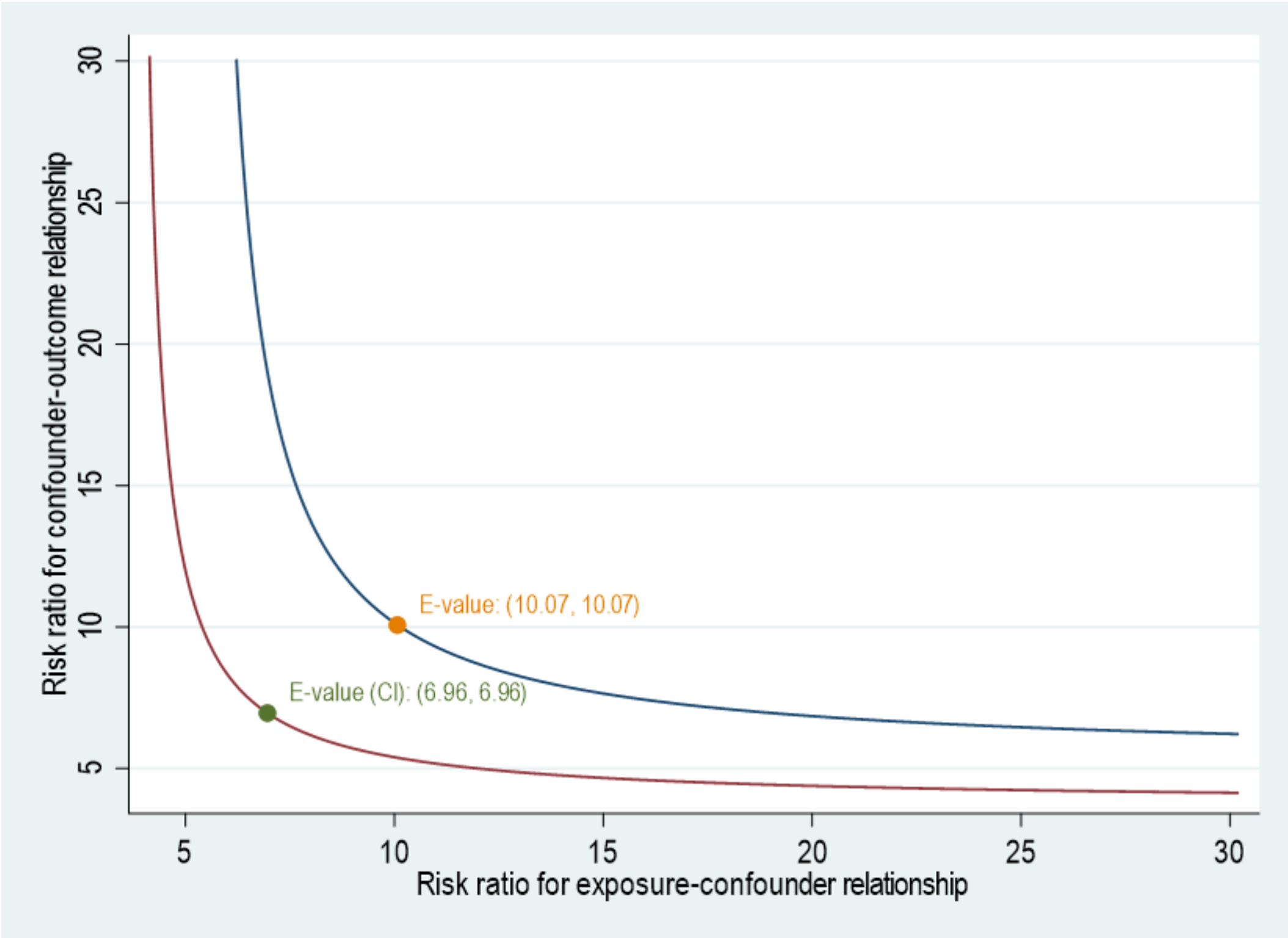

B. Landmark analysis: within 1 month after exit-site infection

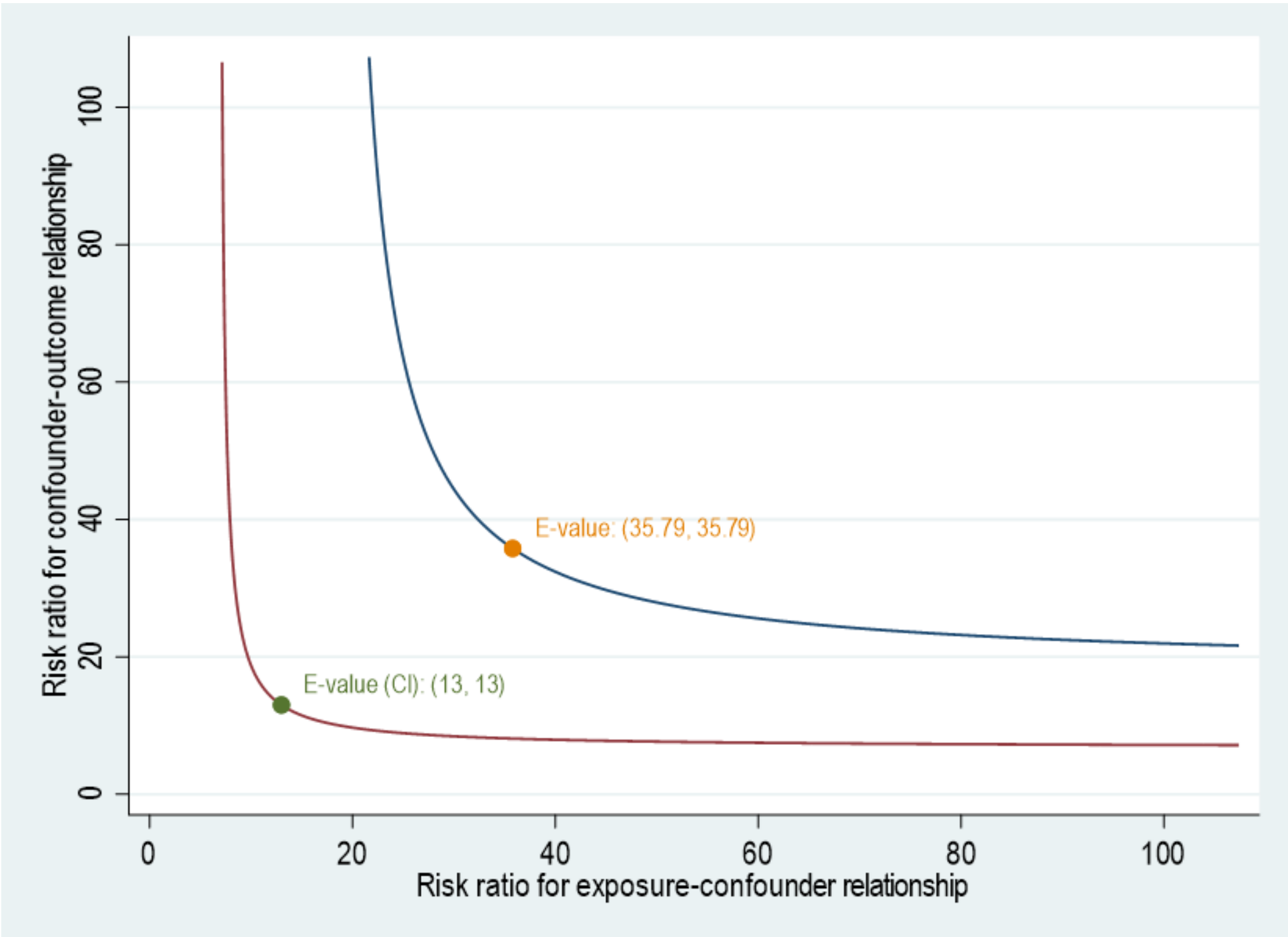

Figure S1 Estimation of E-value (continued)

C. Landmark analysis: 1 – 3 months after exit-site infection

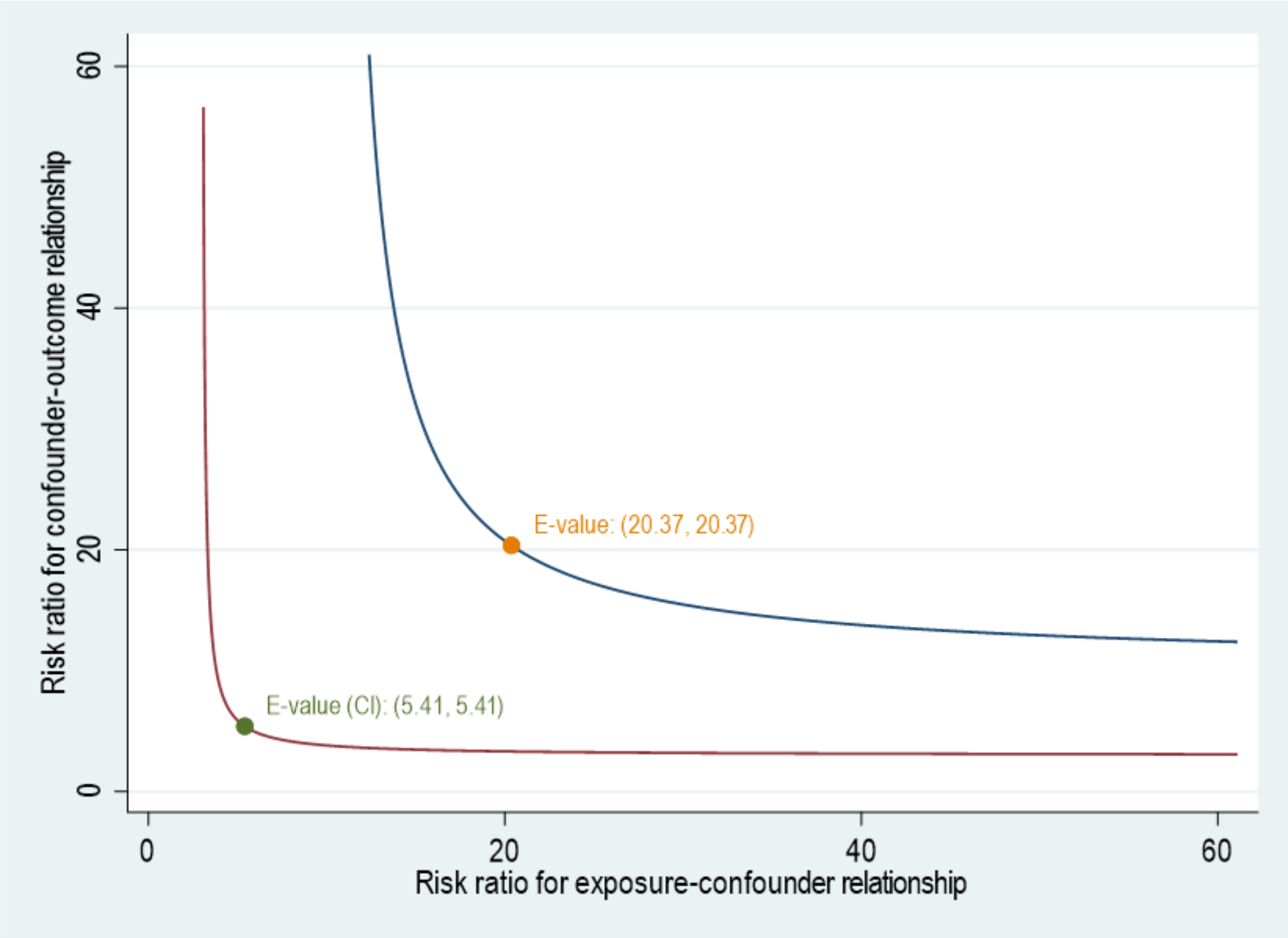

D. Landmark analysis: 3 – 6 months after exit-site infection

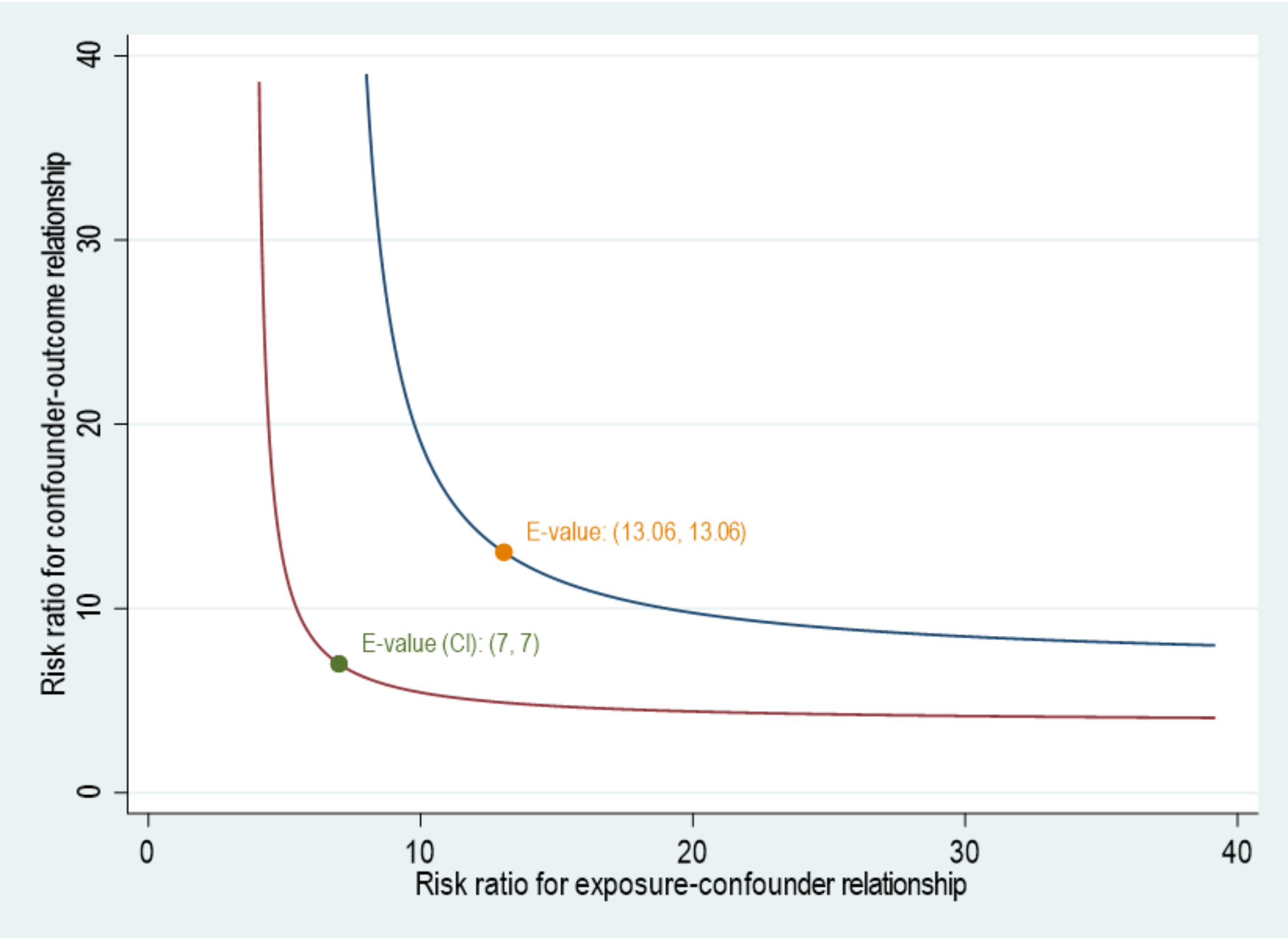

Figure S1 Estimation of E-value (continued)

E. Landmark analysis: 6 – 9 months after exit-site infection

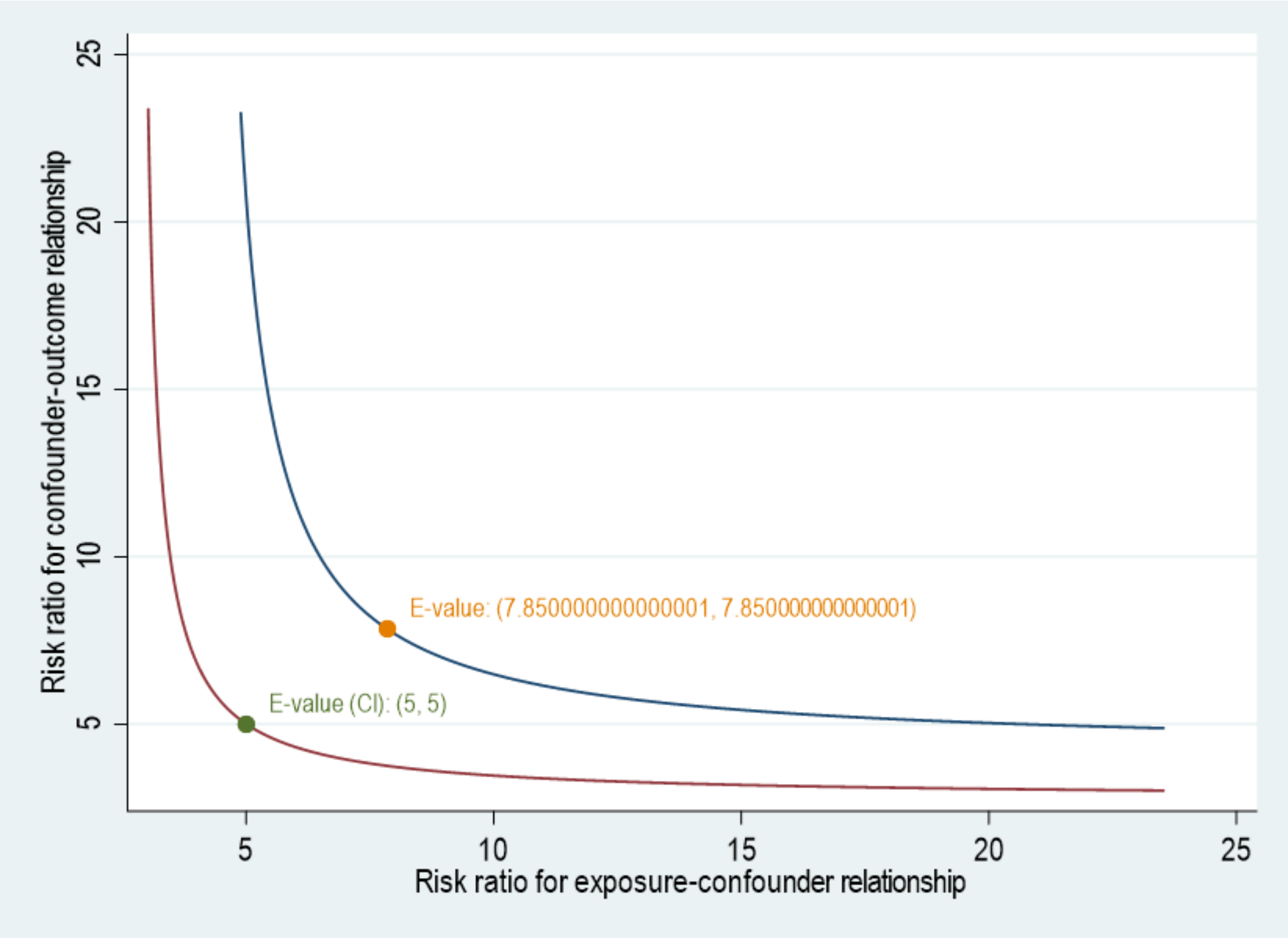

F. Landmark analysis: 9 – 12 months after exit-site infection

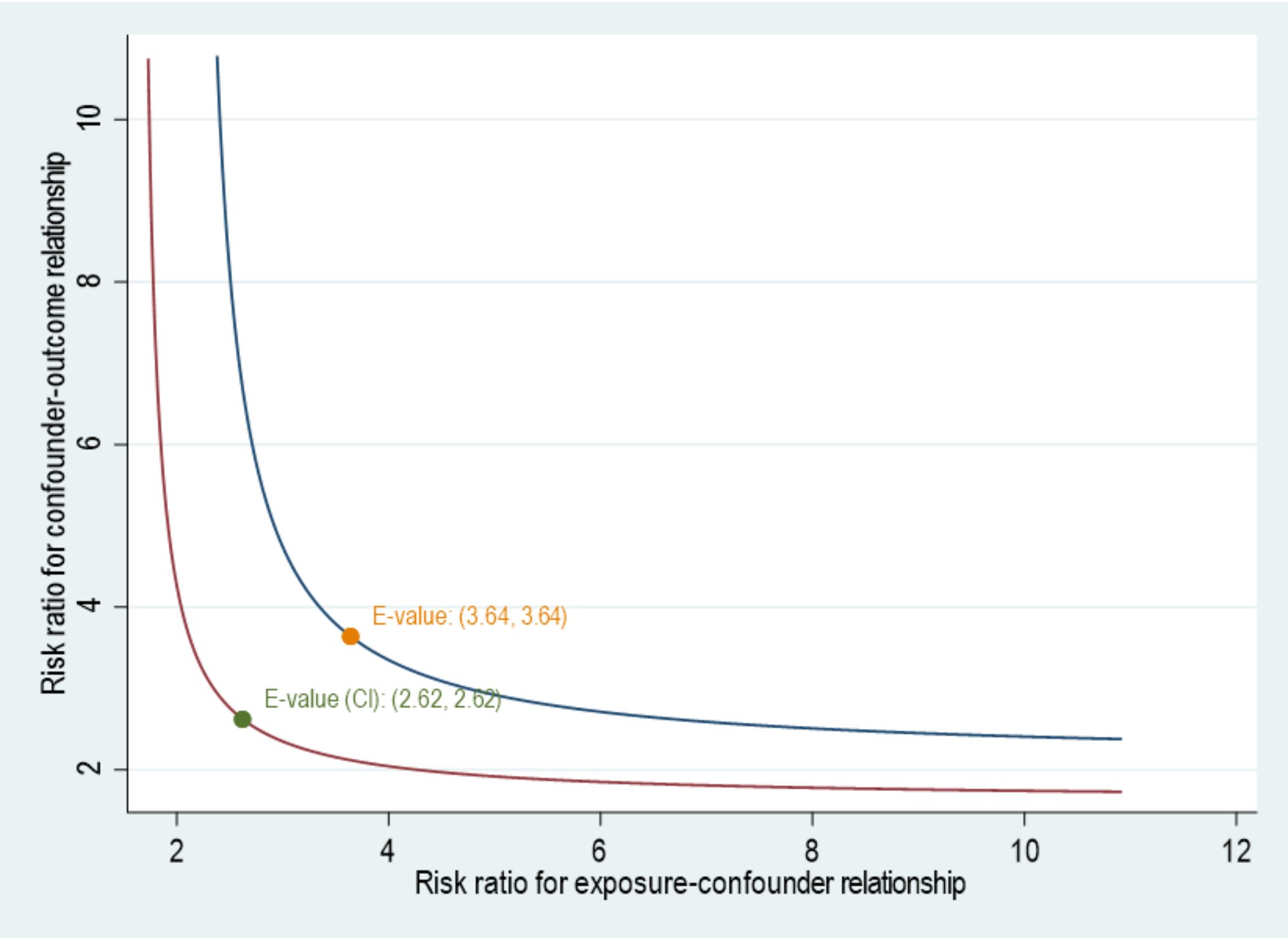

Figure S1 Estimation of E-value (continued)

G. Secondary analysis according to causative organism of exit-site infection: Gram-positive

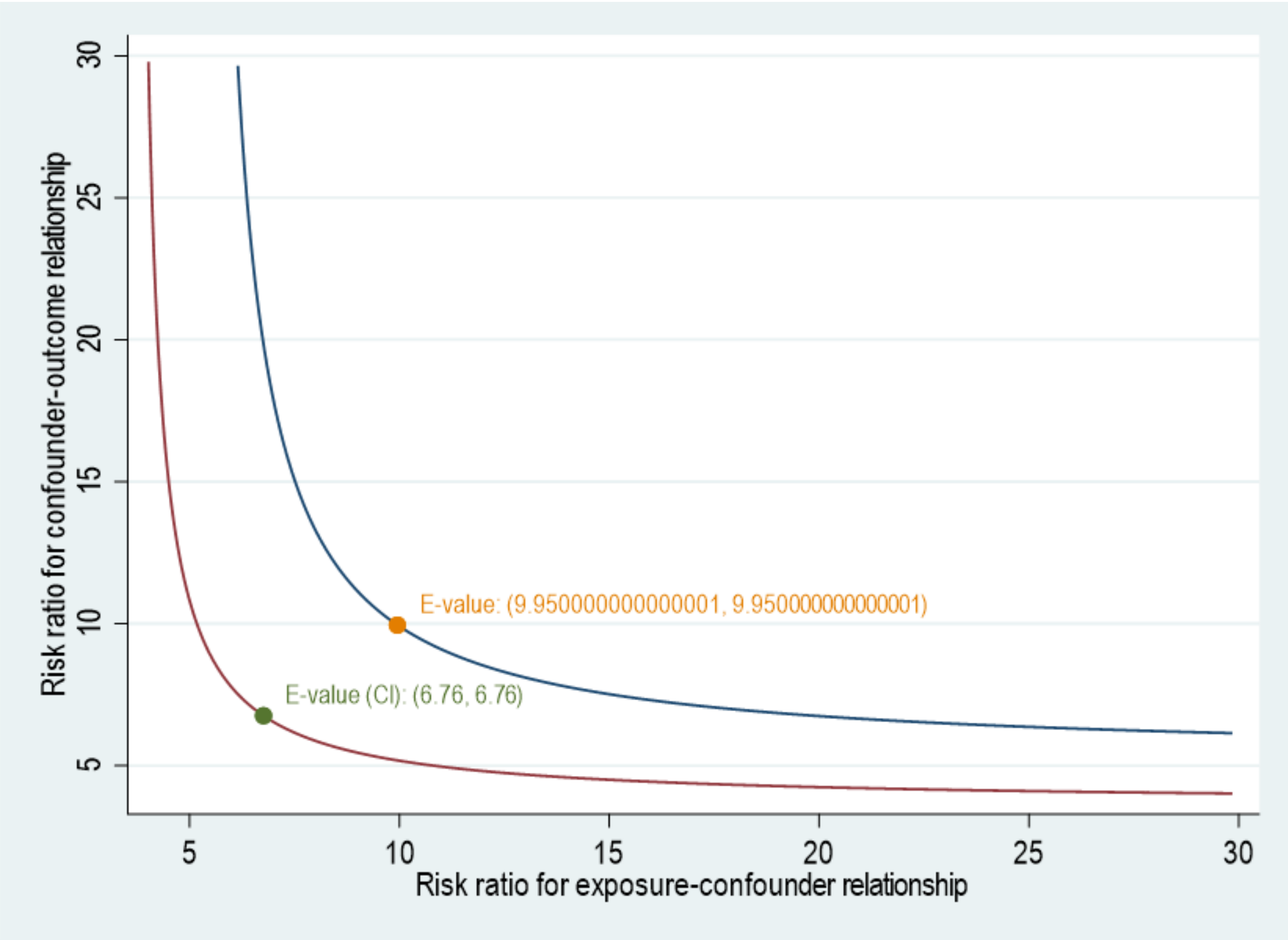

H. Secondary analysis according to causative organism of exit-site infection: Gram-negative

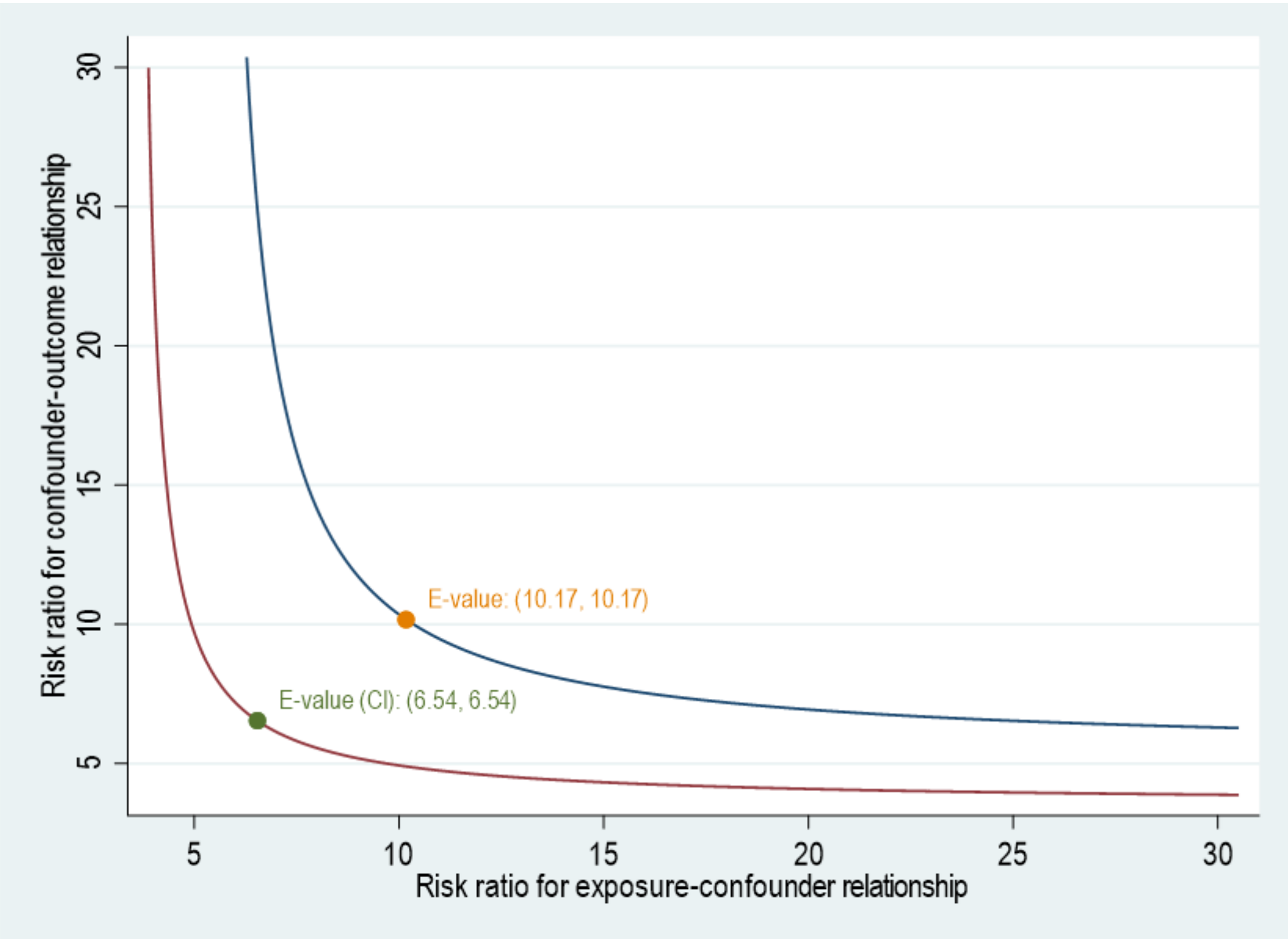

Figure S1 Estimation of E-value (continued)

I. Secondary analysis according to common pathologic organism of exit-site infection: *S. aureus*

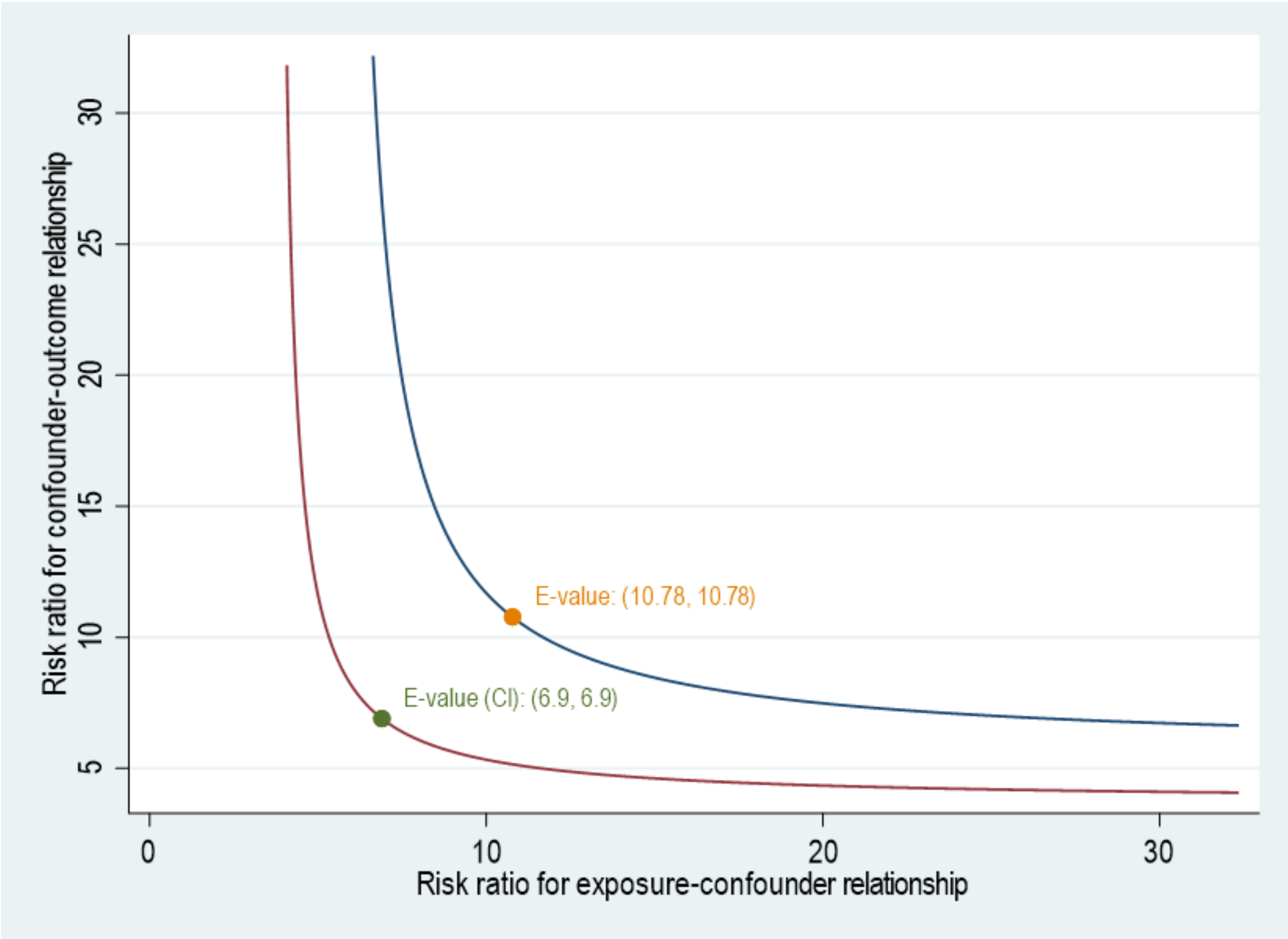

J. Secondary analysis according to common pathologic organism of exit-site infection: CoNS

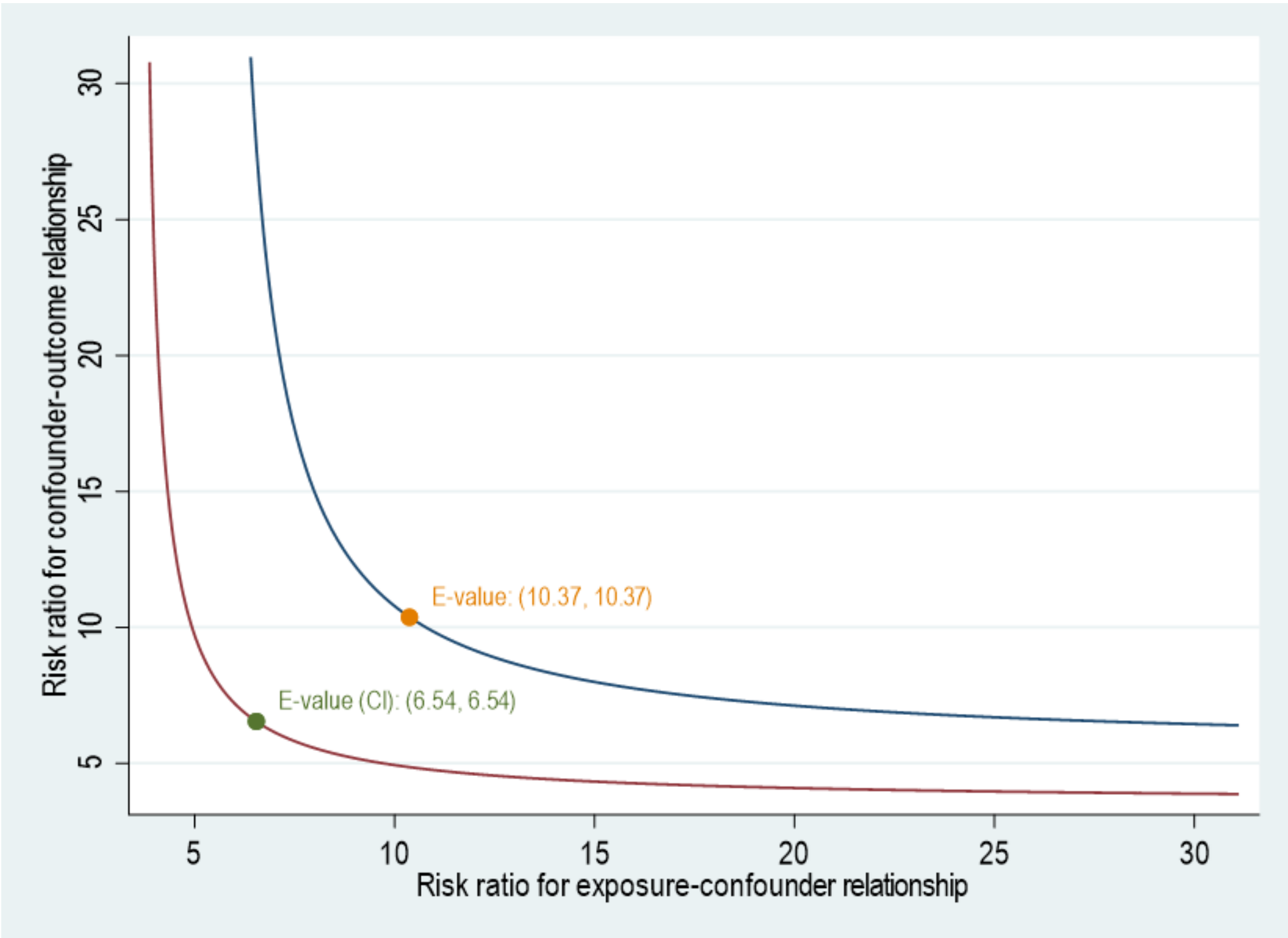

Figure S1 Estimation of E-value (continued)

K. Secondary analysis according to severity strata of causative organism of exit-site infection: low

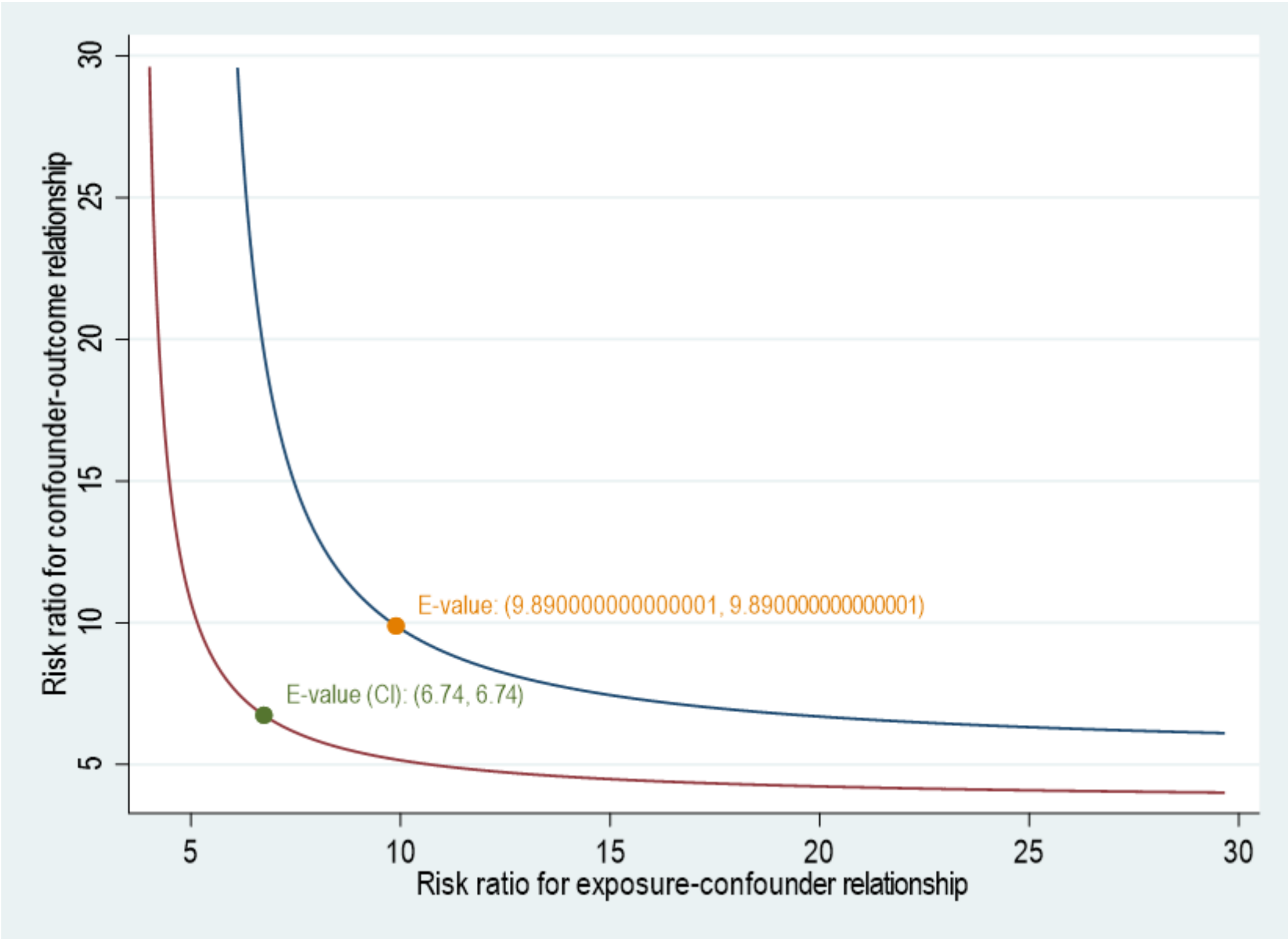

L. Secondary analysis according to severity strata of causative organism of exit-site infection: moderate

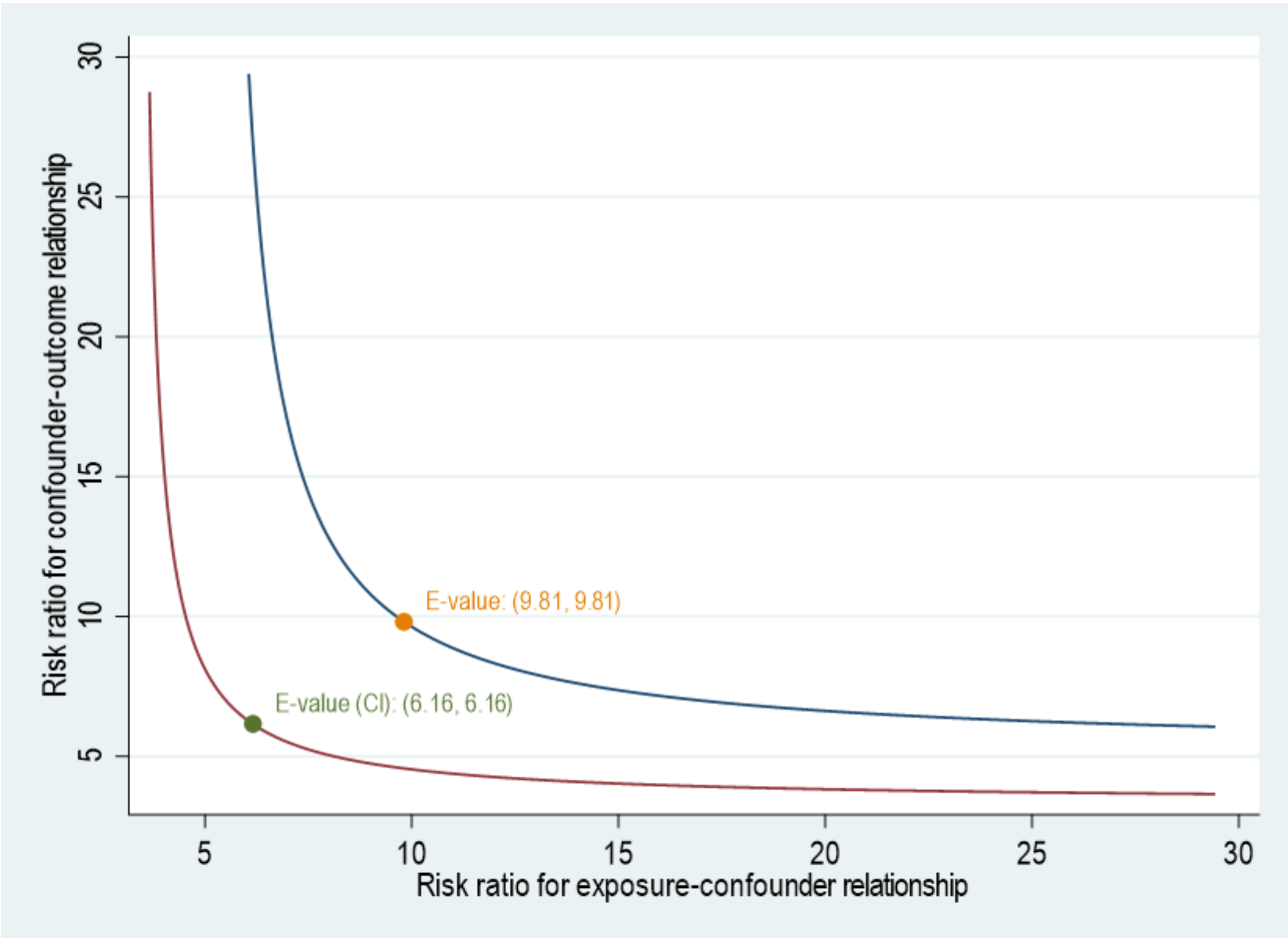

Figure S1 Estimation of E-value (continued)

M. Secondary analysis according to severity strata of causative organism of exit-site infection: high

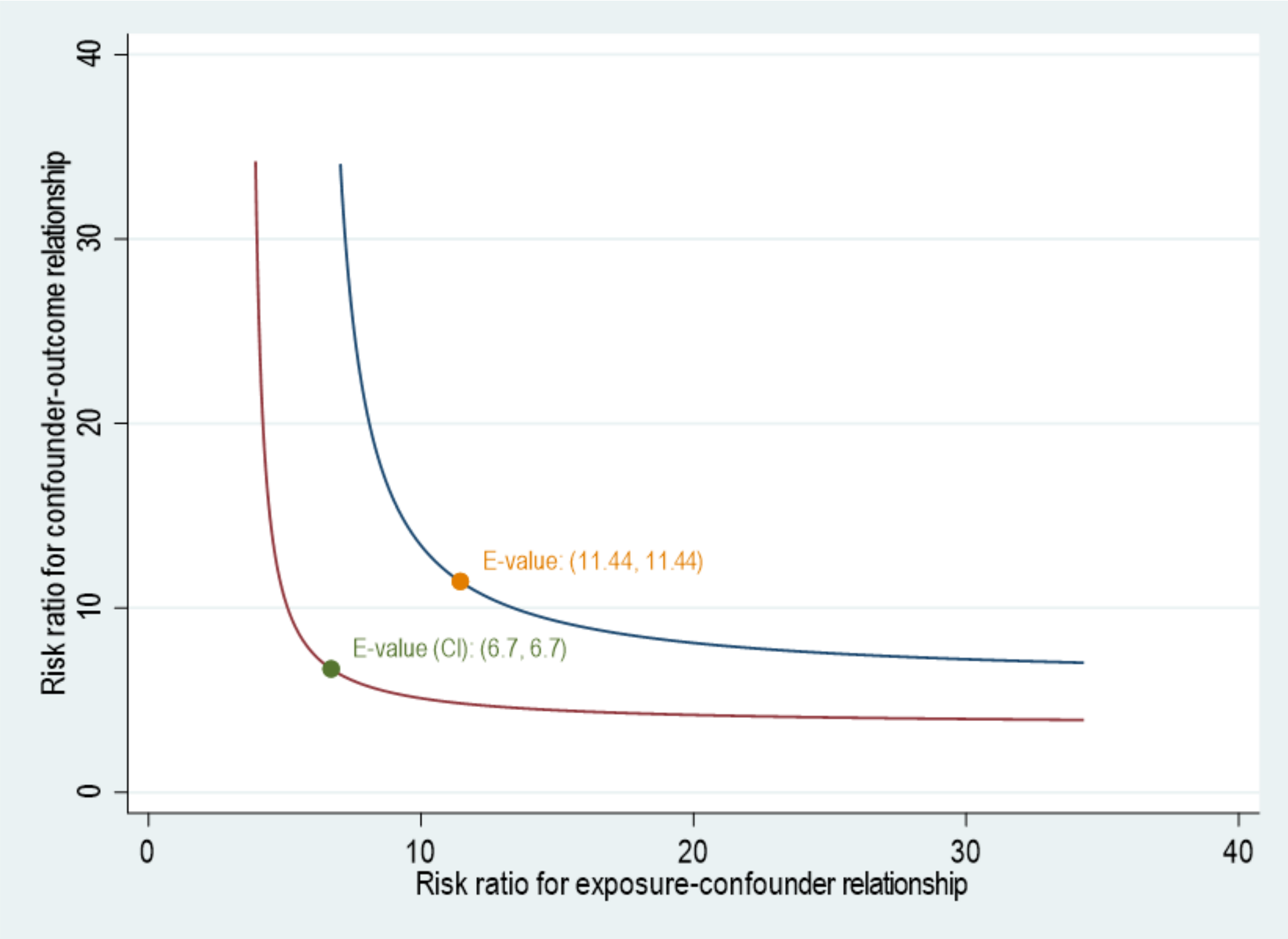

Figure S2 Flowchart of study selection for systematic review and meta-analysis

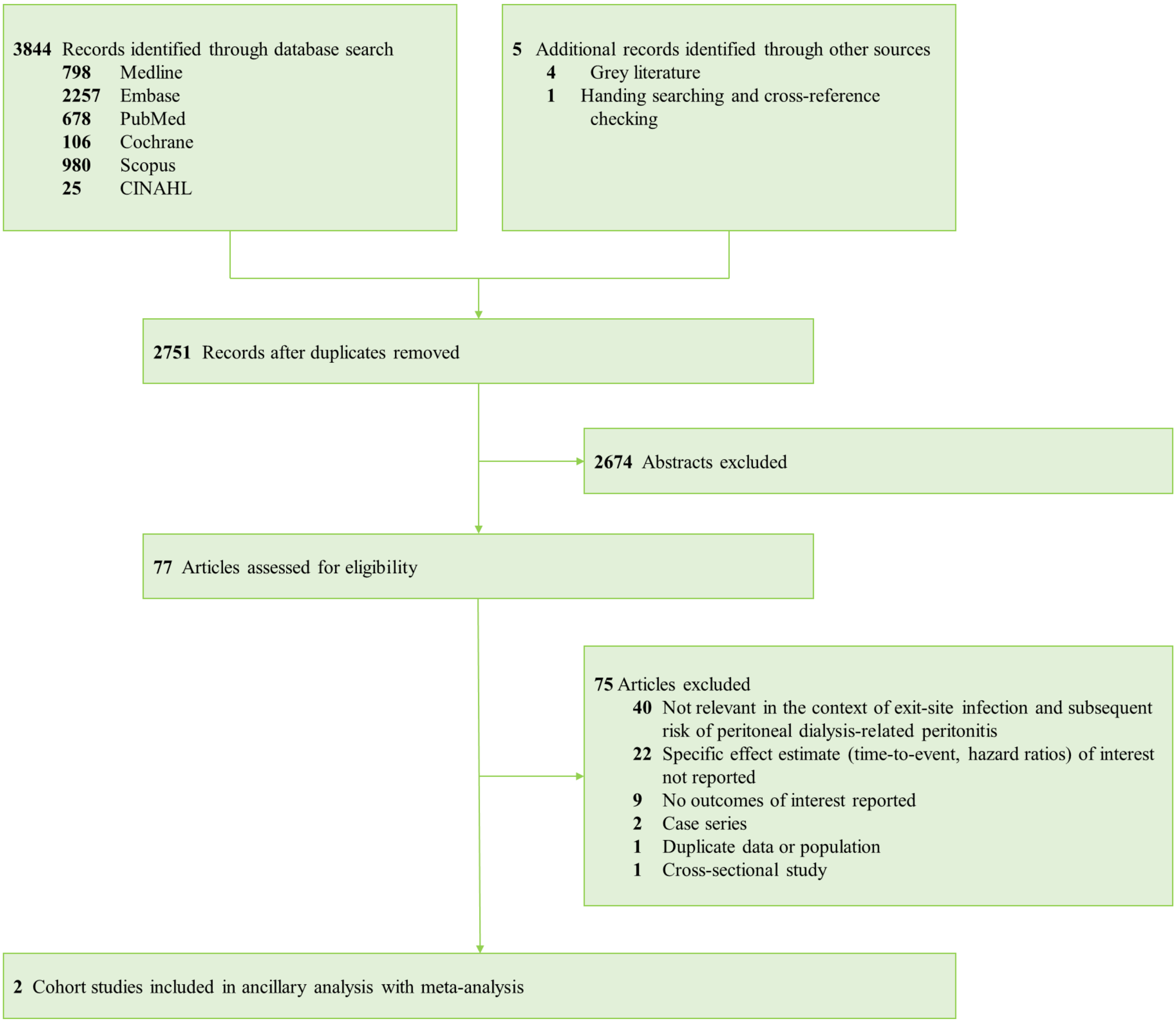

Appendix S1 Methods of ancillary analysis with systematic review and meta-analysis

This ancillary analysis adhered to the Preferred Reporting Items for Systematic Reviews and Meta-Analyses statement.[3] The prespecified protocol was prospectively registered in the International Prospective Register of Systematic Reviews (CRD42018105483).

Data sources and search methods

We searched electronic medical databases, including Medline, Embase, PubMed, Cochrane Library, Scopus, and CINAHL, to identify all relevant articles published from inception to May 25, 2026, without language restrictions. The search strategy was constructed using combinations of Medical Subject Headings, with the main keywords, or a free-text search for exit-site infection and peritoneal dialysis (PD)-associated peritonitis. Details of the search strategy for each major biomedical database are provided as follows:

**Systematic review search strategy**		
**Ovid MEDLINE(R) ALL (1946 to May 25, 2026)**		
**Search**	**Query**	**Items Found**
#1	exp Peritoneal Dialysis/	29685
#2	*dialysis, peritoneal/	16609
#3	(Peritoneal Dialysis or Continuous Ambulatory Peritoneal Dialysis).ti,ab,kf.	31671
#4	exp Continuous Ambulatory Peritoneal Dialysis/	10233
#5	Automated Peritoneal Dialysis.mp	801
#6	(Continuous Ambulatory Peritoneal Dialysis or Automated Peritoneal Dialysis).ti,ab,kf.	7192
#7	or/1-6	38832
#8	Catheter infection$.mp	1432
#9	Exit Site Infection$.mp	1037
#10	Tunnel infection$.mp	511
#11	or/8-10	2721
#12	exp Peritonitis/	29916
#13	(Peritoneum and infection$).ti,ab,kf.	1566
#14	((intraperiton* or periton*) and infection$).ti,ab,kf.	32471
#15	or/12-14	57692
#16	11 and 15	1085
#17	7 and 16	1039
#18	(news or newspaper article or letter or interview comment or editorial or review or systematic review or case report or case series).pt.	6161498
#19	17 not 18	883
#20	limit 19 to humans	798

Systematic review search strategy (continued)		
**Embase (From Inception to May 25, 2026)**		
**Search**	**Query**	**Items Found**
#1	‘peritoneal dialysis’/exp AND [embase]/lim	48868
#2	‘continuous ambulatory peritoneal dialysis’/exp AND [embase]/lim	11203
#3	‘automated peritoneal dialysis’/exp AND [embase]/lim	268
#4	#1 OR #2 OR #3	48868
#5	‘catheter infection*’/exp AND [embase]/lim	22613
#6	(catheter:ti,ab AND infection$:ti,ab) AND [embase]/lim	40049
#7	‘exit site infection*’/exp AND [embase]/lim	1564
#8	‘tunnel infection*’/exp AND [embase]/lim	701
#9	#5 OR #6 OR #7 OR #8	51200
#10	‘peritonitis’/exp AND [embase]/lim	64829
#11	‘peritoneum infection*’/exp AND [embase]/lim	5
#12	((intraperitoneal*:ti,ab OR peritoneal*:ti,ab) AND infection*:ti,ab) AND [embase]/lim	30616
#13	#10 OR #11 OR #12	89649
#14	#9 AND #13	4500
#15	(news:it OR ‘newspaper article’:it OR comment:it OR editorial:it OR interview:it OR letter:it OR review:it OR ‘systematic review’:it OR ‘case report’:it OR ‘case series’:it) AND [embase]/lim	4558729
#16	#14 NOT #15	4044
#17	#16 AND ('article'/it OR 'article in press'/it)	2308
#18	#17 AND ('case control study'/de OR 'clinical article'/de OR 'clinical study'/de OR 'clinical trial'/de OR 'clinical trial topic'/de OR 'cohort analysis'/de OR 'comparative effectiveness'/de OR 'comparative study'/de OR 'control group'/de OR 'controlled clinical trial'/de OR 'controlled study'/de OR 'correlational study'/de OR 'double blind procedure'/de OR 'human'/de OR 'intention to treat analysis'/de OR 'logistic regression analysis'/de OR 'longitudinal study'/de OR 'major clinical study'/de OR 'medical record review'/de OR 'multicenter study'/de OR 'multicenter study topic'/de OR 'non inferiority trial'/de OR 'observational study'/de OR 'open study'/de OR 'outcomes research'/de OR 'parallel design'/de OR 'phase 2 clinical trial'/de OR 'phase 2 clinical trial topic'/de OR 'phase 3 clinical trial'/de OR 'phase 3 clinical trial topic'/de OR 'pilot study'/de OR 'proportional hazards model'/de OR 'prospective study'/de OR 'randomized controlled trial'/de OR 'randomized controlled trial topic'/de OR 'regression analysis'/de OR 'retrospective study'/de OR 'single blind procedure'/de)	2257

Systematic review search strategy (continued)		
**PubMed (From Inception to May 25, 2026)**		
**Search**	**Query**	**Items Found**
#1	(Peritoneal Dialysis) OR (Peritoneal Dialysis[MeSH Terms])	39663
#2	(((((Continuous Ambulatory Peritoneal Dialysis[Title/Abstract]) OR (Automated Peritoneal Dialysis[Title/Abstract])) OR (Continuous Cycling Peritoneal Dialysis[Title/Abstract])) OR (Intermittent Peritoneal Dialysis[Title/Abstract])) OR (Nocturnal Intermittent Peritoneal Dialysis[Title/Abstract])) OR (Tidal Peritoneal Dialysis[Title/Abstract])	7605
#3	#1 OR #2	39663
#4	Exit Site Infection*[Title/Abstract] OR Tunnel infection*[Title/Abstract]	1370
#5	Peritonitis[MeSH Terms]	29910
#6	(intraperiton*[Title/Abstract] OR periton*[Title/Abstract]) AND (infection*[Title/Abstract])	32519
#7	#5 OR #6	57737
#8	#4 AND #7	979
#9	#3 AND #8	971
#10	(((((Case Reports[Publication Type]) OR Guideline[Publication Type]) OR News[Publication Type]) OR Newspaper Article[Publication Type]) OR Review[Publication Type])	6630321
#11	#9 NOT #10	747
#12	Filters applied: Humans	678
**Cochrane Library (From Inception to May 25, 2026)**		
**Search**	**Query**	**Items Found**
#1	MeSH descriptor: [Peritoneal Dialysis] explode all trees	1176
#2	MeSH descriptor: [Peritoneal Dialysis, Continuous Ambulatory] explode all trees	520
#3	(“Continuous Ambulatory Peritoneal Dialysis” OR “Automated Peritoneal Dialysis” OR “Continuous Cycling Peritoneal Dialysis” OR “Intermittent Peritoneal Dialysis” OR “Nocturnal Intermittent Peritoneal Dialysis” OR “Tidal Peritoneal Dialysis”):ti,ab,kw	726
#4	#1 OR #2 OR #3	1454
#5	(Exit Site Infection):ti,ab,kw OR (Exit-Site Infection):ti,ab,kw OR (Tunnel infection):ti,ab,kw	575
#6	MeSH descriptor: [Peritonitis] explode all trees	755
#7	(Intraperiton OR Periton*):ti,ab,kw AND (Infection):ti,ab,kw	1932
#8	#6 OR #7	2399
#9	#5 AND #8	198
#10	#4 AND #9	111
#11	Limit to Trials	106

Systematic review search strategy (continued)		
**Scopus (From Inception to May 25, 2026)**		
**Search**	**Query**	**Items Found**
#1	(TITLE-ABS-KEY (peritoneal AND dialysis) OR TITLE-ABS-KEY (continuous AND ambulatory AND peritoneal AND dialysis) OR TITLE-ABS-KEY (automated AND peritoneal AND dialysis) OR TITLE-ABS-KEY (continuous AND cycling AND peritoneal AND dialysis) OR TITLE-ABS-KEY (intermittent AND peritoneal AND dialysis) OR TITLE-ABS-KEY (nocturnal AND intermittent AND peritoneal AND dialysis) OR TITLE-ABS-KEY (tidal AND peritoneal AND dialysis))	56411
#2	TITLE-ABS-KEY (exit AND site AND infection) OR TITLE-ABS-KEY (tunnel AND infection)	6068
#3	TITLE-ABS-KEY (peritonitis)	78676
#4	TITLE-ABS-KEY (intraperitoneal AND infection) OR TITLE-ABS-KEY (peritoneal AND infection)	42087
#5	#3 OR #4	111925
#6	#2 AND #5	1546
#7	#1 AND #6	1469
#8	Filter: #7 AND (LIMIT-TO (DOCTYPE , "ar")) AND (EXCLUDE (EXACTKEYWORD , "Case Report") OR EXCLUDE (EXACTKEYWORD , "Nonhuman"))	980
**CINAHL (From Inception to May 25, 2026)**		
**Search**	**Query**	**Items Found**
#1	MW peritoneal dialysis	4691
#2	AB "peritoneal dialysis" OR AB "continuous ambulatory peritoneal dialysis" OR AB "automated peritoneal dialysis" OR AB "continuous cycling peritoneal dialysis" OR AB "intermittent peritoneal dialysis" OR AB "nocturnal intermittent peritoneal dialysis" OR AB "tidal peritoneal dialysis"	3323
#3	S1 OR S2	5727
#4	AB "exit site infection" OR "tunnel infection"	125
#5	MW peritonitis	2636
#6	AB "intraperitoneal infection" OR "peritoneal infection"	52
#7	S5 OR S6	2678
#8	S4 AND S7	26
#9	S3 AND S8	26
#10	Expanders - Apply equivalent subjects<br>Source Types - Academic Journals	25

To supplement searches of electronic medical databases, we searched for grey literature on Google Scholar, clinical trial registers, and major nephrology conferences (i.e., American Society of Nephrology [ASN], European Renal Association-European Dialysis and Transplant Association [ERA-EDTA], and International Society of Nephrology [ISN]). Moreover, a manual targeted search of the

reference lists of the included studies, prior systematic reviews, and meta-analyses identified potentially eligible articles.

Study selection and eligibility criteria

After the systematic search, two reviewers (S.N. and C.R.) independently screened the retrieved articles based on their titles and abstracts. Next, potentially relevant full-text articles were abstracted against the study eligibility criteria for the final set of included trials. Any discrepancies during this process were resolved by a third party (K.N. and A.T.).

Details of the study eligibility criteria are described based on the PICOTS framework as follows:

**Study Inclusion and Exclusion Criteria**		
**Category**	**Criteria for inclusion**	**Criteria for exclusion**
Populations (P)	• Patients of all ages received chronic PD (either CAPD or APD) (at least 50 patients)	• In vitro, in vivo, or animal studies<br>• Patients received PD for acute kidney injury<br>• Patients received both PD and HD treatment
Interventions (I) (exposure group)	• Individuals experienced exit-site infection according to the updated 2023 ISPD recommendations[4] (or comparable definitions)	• Unclear definition of exit-site infection
Comparators (C) (non-exposure group)	• Non-exit site infection exposed	• Studies without control groups
Outcomes (O)	• PD-associated peritonitis according to the updated 2022 ISPD recommendations[5] (or comparable criteria)	• Unclear definition of PD-associated peritonitis<br>• Studies with unclear information or insufficient data for recalculating risk estimates (time-to-event, HRs)
Timing (T)	• From the inception of bibliographic databases onward	• Studies were not limited by language or timing frame
Study design (S)	• Published secondary analysis of randomized clinical trials or cohort studies (either retrospective or prospective cohort)	• N-of-one, case series/case reports, non-cohort observational studies (case-control and cross-sectional studies), qualitative studies, narrative reviews, and systematic reviews<br>• Conference abstracts

Abbreviations: APD, automated peritoneal dialysis; CAPD, continuous ambulatory peritoneal dialysis; HRs, hazard ratios; ISPD, PD, peritoneal dialysis.

Data extraction

Two reviewers (S.N. and C.R.) independently extracted the prespecified information based on study and patient characteristics (i.e., study design, country of study conducted, follow-up time, study

size, study population [age, sex, race/ethnicity, body mass index, comorbid conditions], PD modalities (continuous ambulatory [CAPD] or automated peritoneal dialysis [APD]), criteria for diagnosis of exit-site infection and PD-associated peritonitis. Companion studies or secondary analyses of randomized clinical trials (RCTs) that comprised overlapping patients were reassembled. However, when an article reported unclear information or missing data, the corresponding author was contacted to request further clarification.

Study quality assessment

The risk of bias in each included study was assessed using the Newcastle-Ottawa Scale (NOS) for cohort studies[6] by two reviewers (S.N. and C.R.) independently. The overall risk of bias was then categorized as low, high, or of some concern for randomized trials and the highest quality (NOS ≥8 points) for non-randomized studies.[7,8] Any discrepancies at each review step were resolved through consensus.

Statistical analysis

Two additional reviewers (K.N. and A.T.) independently cross-checked and verified the final dataset before formal analysis. Based on the extracted data, we estimated summary hazard ratios (HRs) with 95% confidence intervals (CIs). Summary effect estimates were calculated and presented using random-effects models to account for within- and between-study variance across the included studies. Moreover, we estimated 95% prediction intervals for all pooled effect estimates to account for the range of the true treatment effect in an individual study and the expected uncertainty in the estimate of a new study.[9] We also calculated the expected (E)-value, which accounts for the robustness of the effect estimates between exit-siter infection and subsequent risk of PD-associated peritonitis to potential residual confounders.[10]

Statistical heterogeneity was assessed using Cochran's Q test, with $P<0.05$. The degree of inconsistency was explored using the I^2 index and tau-squared (τ^2) statistics and classified as low (I^2=25.0% and τ^2=0.01), moderate (I^2=50.0% and τ^2=0.06), or high (I^2=75.0% and τ^2=0.16).[11] We

employed funnel plots and tested for statistical publication bias using Egger's tests for each outcome of interest if there were sufficient data (>10 included trials for a particular outcome).[7] A set of subgroup analyses was planned to explore the summary effect estimates based on study characteristics (e.g. study design [retrospective vs prospective cohort], sample size [<100 vs ≥100 patients], geographical location), patient demographics (e.g. age [pediatric/adolescents vs. adults], sex, race/ethnicity, comorbidities [diabetes mellitus, etiology of end-stage kidney disease]), specific microorganisms of exit-site infection and PD-associated peritonitis, and discrete time intervals (risk time period of exit-site infection) in terms of duration-response relationship. We also planned to perform univariable meta-regression to explore the effect modifiers of the meta-analytic estimates using study and patient characteristics.

To address the robustness of the findings, additional sensitivity analyses were planned based on (i) post-hoc analysis using the "leave-one-out" approach (removing individual trials one at a time to assess their impact on summary effect estimates) and (ii) including only studies with the highest quality (NOS ≥8 points).

All statistical analyses and graph visualization were performed using Stata software, version 18.5 (StataCorp LLC, College Station, Texas). Two-tailed *P*-values <0.05 were considered statistically significant.

Certainty assessment and interpretation of evidence findings

For evidence certainty assessment, two reviewers (S.N. and C.R.) independently appraised evidence certainty using the modified recommendations of the United States Agency for Healthcare Research[12] and Quality and the Grading of Recommended Assessment, Development, and Evaluation.[13] Evidence certainty was then classified into insufficient, very low, low, moderate, or high quality.[7] With respect to contextualized approaches of evidence-based conclusions, the pooled association was judged as trivial/neutral (not substantially different from that of the individuals without any episode of exit-site infection), beneficial (protective effect or decreased risk of PD-associated peritonitis), or harmful (increased risk of PD-associated peritonitis).[7,8]

eReferences

1. van Diepen AT, Tomlinson GA, Jassal SV. The association between exit site infection and subsequent peritonitis among peritoneal dialysis patients. *Clin J Am Soc Nephrol*. Aug 2012;7(8):1266-71. doi:10.2215/cjn.00980112
2. Lloyd A, Tangri N, Shafer LA, et al. The risk of peritonitis after an exit site infection: a time-matched, case-control study. *Nephrol Dial Transplant*. Jul 2013;28(7):1915-21. doi:10.1093/ndt/gft002
3. Page MJ, McKenzie JE, Bossuyt PM, et al. The PRISMA 2020 statement: an updated guideline for reporting systematic reviews. *BMJ*. Mar 29 2021;372:n71. doi:10.1136/bmj.n71
4. Chow KM, Li PK, Cho Y, et al. ISPD Catheter-related Infection Recommendations: 2023 Update. *Perit Dial Int*. May 2023;43(3):201-219. doi:10.1177/08968608231172740
5. Li PK, Chow KM, Cho Y, et al. ISPD peritonitis guideline recommendations: 2022 update on prevention and treatment. *Perit Dial Int*. Mar 2022;42(2):110-153. doi:10.1177/08968608221080586
6. Wells G, Shea B, O'Connell D, et al. The Newcastle-Ottawa Scale (NOS) for assessing the quality of nonrandomised studies in meta-analyses. Accessed May 25, 2026, http://www.ohri.ca/programs/clinical_epidemiology/oxford.asp
7. Ngamvichchukorn T, Ruengorn C, Noppakun K, et al. Association Between Pretransplant Dialysis Modality and Kidney Transplant Outcomes: A Systematic Review and Meta-analysis. *JAMA Netw Open*. Oct 3 2022;5(10):e2237580. doi:10.1001/jamanetworkopen.2022.37580
8. Nochaiwong S, Chuamanochan M, Ruengorn C, et al. Use of Thiazide Diuretics and Risk of All Types of Skin Cancers: An Updated Systematic Review and Meta-Analysis. *Cancers (Basel)*. May 23 2022;14(10)doi:10.3390/cancers14102566
9. Riley RD, Higgins JP, Deeks JJ. Interpretation of random effects meta-analyses. *BMJ*. Feb 10 2011;342:d549. doi:10.1136/bmj.d549
10. VanderWeele TJ, Ding P. Sensitivity Analysis in Observational Research: Introducing the E-Value. *Ann Intern Med*. Aug 15 2017;167(4):268-274. doi:10.7326/m16-2607
11. Higgins JP, Thompson SG, Deeks JJ, Altman DG. Measuring inconsistency in meta-analyses. *BMJ*. Sep 6 2003;327(7414):557-60. doi:10.1136/bmj.327.7414.557
12. Berkman ND, Lohr KN, Ansari MT, et al. Grading the strength of a body of evidence when assessing health care interventions: an EPC update. *J Clin Epidemiol*. Nov 2015;68(11):1312-24. doi:10.1016/j.jclinepi.2014.11.023
13. Balshem H, Helfand M, Schünemann HJ, et al. GRADE guidelines: 3. Rating the quality of evidence. *J Clin Epidemiol*. Apr 2011;64(4):401-6. doi:10.1016/j.jclinepi.2010.07.015
